# Task-dependent clustering in permutation-aligned neural network weights

**DOI:** 10.3389/fcogn.2026.1960468

**Published:** 2026-10-26

**Authors:** Patrick Krauss, Achim Schilling, Andreas Maier, Thomas Kinfe, Henri Stübner, Niklas Römmelt, Claus Metzner

**Affiliations:** 1 Cognitive Computational Neuroscience Group, Pattern Recognition Lab, Friedrich-Alexander-University Erlangen-Nürnberg (FAU), Erlangen, Germany; 2 Neuromodulation and Neuroprosthetics, University Hospital Mannheim, University Heidelberg, Mannheim, Germany; 3 Neuroscience Lab, University Hospital Erlangen, Erlangen, Germany; 4 BG Klinik Ludwigshafen, Ludwigshafen am Rhein, Germany

**Keywords:** algorithmic attractors, artificial neural networks, neural network comparison, permutation alignment, representational similarity, task-dependent clustering, training dynamics, weight-space geometry

## Abstract

**Introduction:**

Across biology, physics and computation, complex systems can occupy reproducible regions of state space despite different microscopic histories. Attractor concepts provide one framework for understanding how diverse trajectories may become confined to a limited set of outcomes. Whether stochastic neural-network training similarly organizes models into reproducible, task-dependent regions is difficult to test because functionally equivalent networks can differ through hidden-unit permutations and rectified linear unit (ReLU) rescalings.

**Methods:**

Here, we followed independently initialized shallow networks trained on several image-classification problems and aligned their hidden units before comparing their weights.

**Results:**

Networks trained on the same dataset developed more similar weight structure than networks trained on different datasets. During the first few stochastic gradient descent (SGD) updates, accuracy improved sharply while displacement from initialization remained small. These small movements were not structurally neutral: dataset-dependent separation emerged almost immediately in both aligned weights and hidden representations. Changing the prediction rule while holding the input images fixed produced a corresponding task-dependent separation, showing that the organization reflected the learning objective rather than the image distribution alone. The principal finding persisted after removing ReLU scaling freedom. Git Re-Basin yielded stronger neuronal correspondence than our independently developed Hungarian procedure, although the latter still recovered functionally meaningful alignments.

**Discussion:**

Together, the results show that training can rapidly organize small parameter changes into dataset- and task-dependent clusters. The effectiveness of small early updates also motivates a target-rich landscape hypothesis in which useful solution regions are locally accessible despite non-unique parameterizations.

## 1 Introduction

Artificial neural networks offer a useful meeting point between artificial intelligence and the study of cognition. They are not literal replicas of nervous systems. Instead, they turn hypotheses about learning and information processing into explicit mechanisms that can be trained, perturbed, and measured at every level. This makes them valuable as controlled model systems: a network can solve a perceptual or cognitive problem, while its internal activity and every change in its connections remain accessible. In this sense, neural networks can help connect behavioral questions to computational mechanisms, complementing analyses at Marr's computational and algorithmic levels (Marr, 1982). Their rapid development in machine learning has widened the range of functions that can be studied in this way (LeCun et al., 2015; Alzubaidi et al., 2021).

Two broad classes emphasize different aspects of cognition. Feed-forward networks transform an input through a sequence of layers and are especially useful for studying perception, categorization, and the gradual formation of abstract features. Recurrent networks feed activity back into the system. They can therefore maintain internal state, combine current input with prior context, and produce memory-like or attractor-like dynamics. Our own work has used both perspectives. We introduced the Generalized Discrimination Value to follow how class separability changes across network layers (Schilling et al., 2021); studied how recurrent and reservoir networks organize nonlinear dynamics and robust functional regimes (Metzner and Krauss, 2022; Metzner et al., 2025a,b); examined successor representations as a bridge between neural computation and cognitive maps (Stoewer et al., 2022); and investigated how recurrent and transformer language models encode linguistic categories and relations (Krauss et al., 2025; Ramezani et al., 2025; Kissane et al., 2026; Kölbl et al., 2026). These studies ask what a trained network represents or computes. They lead naturally to a more basic structural question: when several networks learn the same problem independently, do they also arrive at related internal organizations?

At first sight, this question seems easy to answer. We can train several copies of the same architecture and compare their weights. Yet the coordinates of a neural network do not uniquely identify its function. Hidden neurons can be permuted if the corresponding incoming and outgoing weights are permuted together. In networks with positively homogeneous activations such as rectified linear unit (ReLU), one can also multiply the incoming weights of a hidden neuron by a positive factor and divide its outgoing weights by the same factor without changing the computed function. Two networks may therefore implement closely related solutions while appearing far apart in a direct, coordinate-wise comparison.

These symmetries mean that a functional solution is represented by an equivalence class rather than an isolated parameter vector. This motivates the target-rich landscape hypothesis: symmetry-related realizations and additional nearly equivalent solutions could make useful regions accessible through small coordinated updates. The hypothesis concerns accessibility of solutions, not an established attractor property.

This ambiguity matters because independently initialized networks usually assign different indices and scales to otherwise corresponding features. A large raw weight distance could thus mean that the networks learned different structures, or merely that the same structure is written in different coordinates. Permutation-aware studies have made this distinction explicit and shown that suitable neuron assignments can reveal connections between solutions that are hidden in the original numbering (Entezari et al., 2022; Ainsworth et al., 2023). To study whether training produces reproducible weight structure, one must first compare networks only after accounting for these known symmetries.

Removing coordinate ambiguity still leaves several meanings of similarity. Networks converge functionally when they make similar predictions or reach similar accuracy. This is the most visible and, for networks trained on the same task, the most expected form of agreement. However, equal performance does not require equal mechanisms: many internal computations can support the same input–output mapping. Networks may instead converge at the level of hidden representations, encoding related distinctions even if individual neurons differ. Most stringently, they may also develop similar organizations in parameter space after symmetry alignment.

These levels need not coincide. Functional similarity could arise from different representations, and representational similarity could survive transformations that leave no simple parameter-wise correspondence. Conversely, small but coordinated weight changes could alter representations and behavior long before the network has moved far from its initialization. The rapid rise of accuracy early in training makes this last possibility especially relevant. High early performance could mean that only a weak, largely generic adjustment is needed, or that small movements already point in task-specific directions. We therefore treat performance, hidden representations, and aligned weights as complementary observations rather than interchangeable measures.

Previous work has shown that independently trained networks can learn partly shared features and representations (Li et al., 2015). Methods such as singular vector canonical correlation analysis (SVCCA), projection-weighted canonical correlation analysis (CCA), and centered kernel alignment (CKA) compare activation spaces without requiring a one-to-one match between neuron indices (Raghu et al., 2017; Morcos et al., 2018; Kornblith et al., 2019). More recent work has extended these comparisons across architectures, tasks, and model families, and has raised the possibility that increasingly capable systems may approach common representational structures (Csiszárik et al., 2021; Klabunde et al., 2025; Huh et al., 2024).

This literature makes representational agreement plausible, but it also sharpens the unresolved question. Similar activation geometry could reflect a common computation implemented by structurally different weights, whereas aligned weight similarity would point to a more constrained solution. A weight-space pattern, in turn, could be created by the chosen alignment or distance measure rather than by functionally meaningful correspondence. To separate these possibilities, we compare aligned weights and hidden representations in parallel and validate inferred neuron matches using activation correlations that were not used to construct the assignments.

Permutation-aware weight matching provides the necessary bridge from unordered hidden units to comparable parameter coordinates. Git Re-Basin formulates this problem as the search for permutations that increase agreement between the complete weight tensors of two networks, and it has become a strong reference method for aligning independently trained models (Ainsworth et al., 2023). Related work has shown why permutation symmetries can hide simple relations between solutions (Entezari et al., 2022).

We also evaluate a Hungarian-assignment procedure based on permutation-invariant features (PIFs). The central idea was developed in our group and documented in two Master's theses submitted in 2019, but it was not published in an archival research venue. We therefore introduce it here as a previously documented and interpretable alignment variant, not as a claim of priority, general novelty, or superiority over established methods. Because any apparent clustering could depend on how neurons are paired, comparing PIF-Hungarian matching with Git Re-Basin is essential. If both methods recover meaningful correspondence but one does so more strongly, the shared pattern is more credible and the difference helps define the practical limits of the earlier procedure.

A broader analogy comes from convergent evolution. In biology, different histories can produce similar forms when organisms face related constraints and selection pressures (Conway Morris, 2003; Losos, 2017; Petryshen et al., 2020). Learning systems also combine historical contingency with constraint: their initial weights and stochastic update sequences differ, but architecture, data, objective, and optimizer restrict which solutions work well. This suggests that repeated training may not fill parameter space uniformly. Instead, it may guide networks toward a limited set of task-dependent regions.

Attractor language offers an intuitive way to describe this possibility. If many trajectories enter a reproducible region despite different starting points, that region resembles an algorithmic attractor. Yet endpoint clustering alone cannot establish an attractor, because it neither maps a basin of attraction nor tests recovery after perturbation. The analogy therefore motivates a measurable first question rather than predetermining its answer: after known symmetries are removed, do independent training trajectories form reproducible clusters that depend on the data and the task?

We address this question in a sequence of controlled comparisons. First, we ask whether networks trained on the same image dataset become more similar in aligned weight space than networks trained on different datasets. A positive result could arise from the input distributions, from the learned classification rules, or from both. We therefore hold the images fixed and change only the target rule to test whether the objective itself redirects the emerging structure.

Next, we ask whether weight-space clustering has a counterpart in hidden activity. We compare aligned weights with linear CKA and use matched-neuron activation correlations as an independent check of assignment quality. Because raw parameter distances can also reflect the positive scaling freedom of ReLU units, we repeat the principal comparison in a canonical gauge. We then compare PIF-Hungarian matching directly with Git Re-Basin to determine which conclusions survive a change in alignment method.

Finally, endpoint comparisons cannot show when structure appears. We therefore resolve the earliest stochastic-gradient updates and follow accuracy, displacement from initialization, aligned weight structure, and hidden representations together. This analysis asks whether dataset-dependent organization begins only after the network has traveled far through parameter space, or whether modest early changes already carry a specific direction. Together, these tests distinguish reproducible task-dependent clustering from stronger claims of identical mechanisms, universal solutions, or demonstrated attractor dynamics.

## 2 Materials and methods

### 2.1 Study design and analysis overview

We used a sequence of controlled experiments to ask whether independently initialized neural networks acquire reproducible weight structure during training. The main experiment compared networks trained on the Modified National Institute of Standards and Technology (MNIST) dataset, Fashion-MNIST, and Kuzushiji-MNIST (KMNIST). Because these datasets differ in both their image distributions and their class semantics, we refer to this first comparison as a *dataset* comparison. A separate paired experiment used exactly the same MNIST images with two different target rules, digit identity and parity, to isolate a *task* effect at fixed input distribution.

The main cohort comprised 30 independent training runs, 10 for each dataset. At each saved checkpoint, every unordered pair of networks was compared after hidden-neuron alignment. Weight-space analyses were performed separately for the input-to-hidden matrix *W*_0_ and the hidden-to-output matrix *W*_1_, and were also summarized by layer-balanced and parameter-count-weighted averages. The primary weight-space outcome was canonically gauged, layer-balanced Alpha dissimilarity with α = 2 at the final checkpoint. It used direct canonical-Alpha profile Hungarian alignment within our PIF/Hungarian framework; Git Re-Basin served as an external benchmark rather than a replacement primary analysis. Raw Alpha quantified sensitivity to ReLU rescaling, while Euclidean dissimilarity and Structural Network Dissimilarity (SND) served as complementary measures.

Three complementary analyses tested whether the weight-space result generalized beyond its own measurement procedure. First, hidden representations were compared with linear CKA. Second, the quality of the inferred neuron assignments was evaluated by the correlation of matched hidden-neuron activations. Third, our alignment was compared with the established Git Re-Basin weight-matching objective. A densely checkpointed SGD experiment resolved the onset of dataset-dependent structure during the first two epochs. Analysis plans specifying endpoints, comparison groups, and resampling units were saved before the corresponding computations. The unit of replication was always a complete training run or a paired seed block, never an individual network pair.

### 2.2 Datasets and preprocessing

We used MNIST, Fashion-MNIST, and KMNIST. Each dataset contains 60,000 training and 10,000 test images, with 10 nominal classes. Images are grayscale arrays of 28 × 28 pixels and were flattened to 784-dimensional input vectors. MNIST contains handwritten digits, Fashion-MNIST contains clothing categories, and KMNIST contains cursive Japanese characters. No data augmentation was applied, and the published training and test partitions were left unchanged.

Pixel intensities were converted to floating point, divided by 255, and standardized with the conventional dataset-specific constants. The means and standard deviations were (0.1307, 0.3081) for MNIST, (0.2860, 0.3530) for Fashion-MNIST, and (0.1918, 0.3483) for KMNIST. The locally cached, uncompressed IDX files were read directly. This bypassed an incompatibility between the installed versions of PyTorch and torchvision without changing the numerical input. A complete cross-check against the framework-based data loader produced identical weights at every checkpoint.

### 2.3 Network architecture and training

The principal architecture was a fully connected multilayer perceptron, as specified in Equation 1:


$$\begin{array}{l} 784\to 64\to 10. \end{array}$$ (1)

For a row-vector input *x*, its logits were given by Equation 2:


$$\begin{array}{l} f\left(x\right)=\mathrm{ReLU}\left(xW_{0}\right)W_{1}, \end{array}$$ (2)

where $W_{0}\in {\mathbb{R}}^{784\times 64}$ and $W_{1}\in {\mathbb{R}}^{64\times 10}$. Both linear layers were initialized with the PyTorch default initializer. Biases were set to zero, excluded from optimization, and checked after each update; consequently, all structural analyses involved only *W*_0_ and *W*_1_.

Networks were trained in 32-bit floating point by minimizing cross-entropy loss with stochastic gradient descent (SGD), a learning rate of 0.1, momentum 0.9, and mini-batches of 256 images. Training lasted 10 complete epochs. We use the episode index *E* for half-epoch checkpoints: *E* = 0 is initialization, *E* = 1 is the first half epoch, and *E* = 20 is the end of epoch 10. We saved weights, test loss, and test accuracy at all 21 checkpoints *E* = 0, …, 20. Training examples were reshuffled at every epoch with a deterministic generator derived from the run seed. Test data were never shuffled.

### 2.4 Main training cohort

The main cohort contained 10 independently initialized networks per dataset: M1–M10 for MNIST with seeds 101–110, F1–F10 for Fashion-MNIST with seeds 201–210, and K1–K10 for KMNIST with seeds 301–310. All 30 runs used the architecture, preprocessing, optimizer, and checkpoint schedule described above. Thus, each analysis checkpoint contained 30 × 29/2 = 435 unordered network pairs. Of these, 135 pairs joined networks trained on the same dataset and 300 joined networks trained on different datasets.

Before analysis, every run was required to contain all 21 checkpoints, finite arrays of shapes 784 × 64 and 64 × 10, the intended seed, and a complete test-performance history. File-level Secure Hash Algorithm 256-bit (SHA-256) hashes were recorded in a cohort manifest. Pairwise calculations were checked against independently recomputed subsets of the cohort.

### 2.5 Permutation symmetry of hidden neurons

The numerical labels of hidden neurons are arbitrary. Let *P* be a 64 × 64 permutation matrix and define the equivalent parameterization in Equation 3:


$$\begin{array}{l} W_{0}^{\prime }=W_{0}P,\;W_{1}^{\prime }=P^{T}W_{1}. \end{array}$$ (3)

Because ReLU acts elementwise and commutes with permutations, the network function is preserved, as shown in Equations 4–6:


$$\begin{array}{ll} \mathrm{ReLU}\left(xW_{0}^{\prime }\right)W_{1}^{\prime } & =\mathrm{ReLU}\left(xW_{0}P\right)P^{T}W_{1} \end{array}$$ (4)

$$\begin{array}{l} =\mathrm{ReLU}\left(xW_{0}\right)PP^{T}W_{1} \end{array}$$ (5)

$$\begin{array}{l} =\mathrm{ReLU}\left(xW_{0}\right)W_{1}. \end{array}$$ (6)

Directly comparing unaligned weight entries can therefore assign a large distance to two parameterizations that implement the same function. In all permutation-aware comparisons, network A was kept fixed and a single shared permutation was applied to the columns of *W*_0, *B*_ and the corresponding rows of *W*_1, *B*_.

### 2.6 PIF-initialized metric-consistent Hungarian matching

Our alignment procedure combines a coarse initialization based on permutation-invariant features (PIFs) with Hungarian assignment based on complete neuron profiles. For hidden neuron *i*, let its incoming and outgoing weights be the vectors defined in Equation 7:


$$\begin{array}{l} u_{i}=W_{0}\left[:,i\right],\;v_{i}=W_{1}\left[i,:\right] \end{array}$$ (7)

denote its incoming and outgoing weights. Its eight-dimensional PIF vector was defined as in Equation 8:


$$\begin{array}{l} \phi_{i}=(\sum u_{i},\;\#\{u_{i}\neq 0\},\;\#\{u_{i}>0\},\;\|u_{i}{\|}_{2},\\ \;\sum v_{i},\;\#\{v_{i}\neq 0\},\;\#\{v_{i}>0\},\;\|v_{i}{\|}_{2}). \end{array}$$ (8)

For each network pair, every PIF dimension was standardized using the pooled mean and standard deviation across the 128 neurons of both networks. Euclidean distances between the standardized PIF vectors formed a 64 × 64 cost matrix, and the Hungarian algorithm supplied an initial permutation. The nonzero counts are constant in the dense networks studied here, but were included to keep the definition applicable to sparse networks.

The refinement step used the full local neuron profile defined in Equation 9:


$$\begin{array}{l} q_{i}=\left(u_{i},v_{i}\right) \end{array}$$ (9)

and the same local dissimilarity that was subsequently used to compare the aligned matrices. For a selected measure *d*, the assignment cost was *C*_*ij*_ = *d*(*q*_*i, A*_, *q*_*j, B*_). The Hungarian solution was then applied consistently to both adjacent matrices. For a network with one hidden layer this is a single linear assignment over fixed profiles; in the general multilayer implementation, neighboring layers are revisited in backward and forward zig-zag sweeps until the orders stop changing, the best network dissimilarity improves by less than 10^−12^, or 30 passes have been completed. PIF initialization was enabled for the Euclidean, SND, and raw Alpha main computations. In the vectorized canonical-Alpha calculation, the direct one-hidden-layer assignment was used because PIF presorting can only permute the columns of the same fixed profile-cost matrix and cannot change its Hungarian optimal objective value; this does not require identical assignments when optima are tied. Each measure used its own metric-consistent cost matrix; an alignment inferred with one measure was not silently reused for another. Here, metric-consistent means that the local profile costs use the selected dissimilarity definition. For Alpha, independently normalized profile costs do not imply minimization of the resulting matrix-level Alpha.

In the Git Re-Basin baseline comparison, we evaluated both the PIF assignment and the direct canonical-Alpha profile assignment under the final parameter-count-weighted Alpha objective and selected the lower-dissimilarity candidate. The same deterministic rule was used for every network pair and checkpoint.

We refer to this documented benchmark rule as candidate-selected PIF/Alpha-profile Hungarian alignment, distinguishing it from the direct canonical-Alpha profile Hungarian alignment in Figures 1, 2. The benchmark selection objective is parameter-count-weighted, whereas the displayed Alpha endpoint is layer-balanced. Selecting a lower value under the former objective need not lower the latter. The endpoints in Figures 1, 3 are therefore defined by different assignment and selection procedures and should not be read as duplicate estimates from an identical alignment. Agreement of group means to four decimal places does not imply identical pairwise assignments.

**Figure 1:**
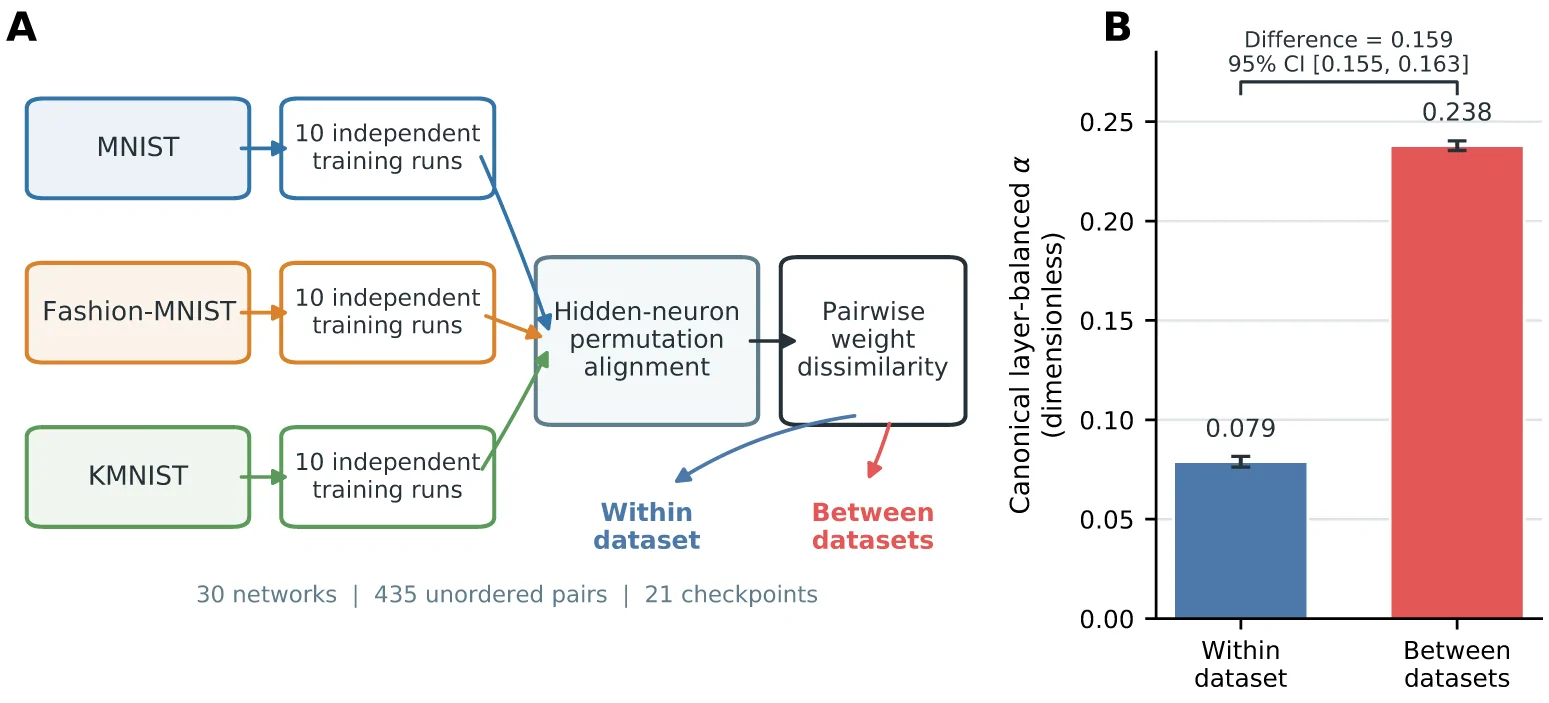
Permutation-aligned networks reveal dataset-dependent weight-space clustering. **(A)** Thirty independently initialized networks were trained on MNIST, Fashion-MNIST, or KMNIST (10 runs per dataset). At each of 21 checkpoints, hidden neurons were aligned by permutation before all 435 unordered network pairs were compared. Pairwise dissimilarities were separated into comparisons within the same dataset and comparisons between datasets. **(B)** Canonically gauged, layer-balanced Alpha dissimilarity (α = 2) at the final checkpoint. A shared permutation was inferred by direct canonical-Alpha profile Hungarian assignment using incoming and outgoing weights, then *W*_0_ and *W*_1_ Alpha were averaged equally. Bars show group estimates and error bars show 95% confidence intervals obtained by a dataset-stratified delete-one-run jackknife. Mean dissimilarity was 0.0789 within datasets and 0.2379 between datasets, yielding a between-minus-within contrast of 0.1590 (95% confidence interval, 0.1553–0.1627). The independent unit for uncertainty estimation was a complete training run rather than an individual network pair.

**Figure 2:**
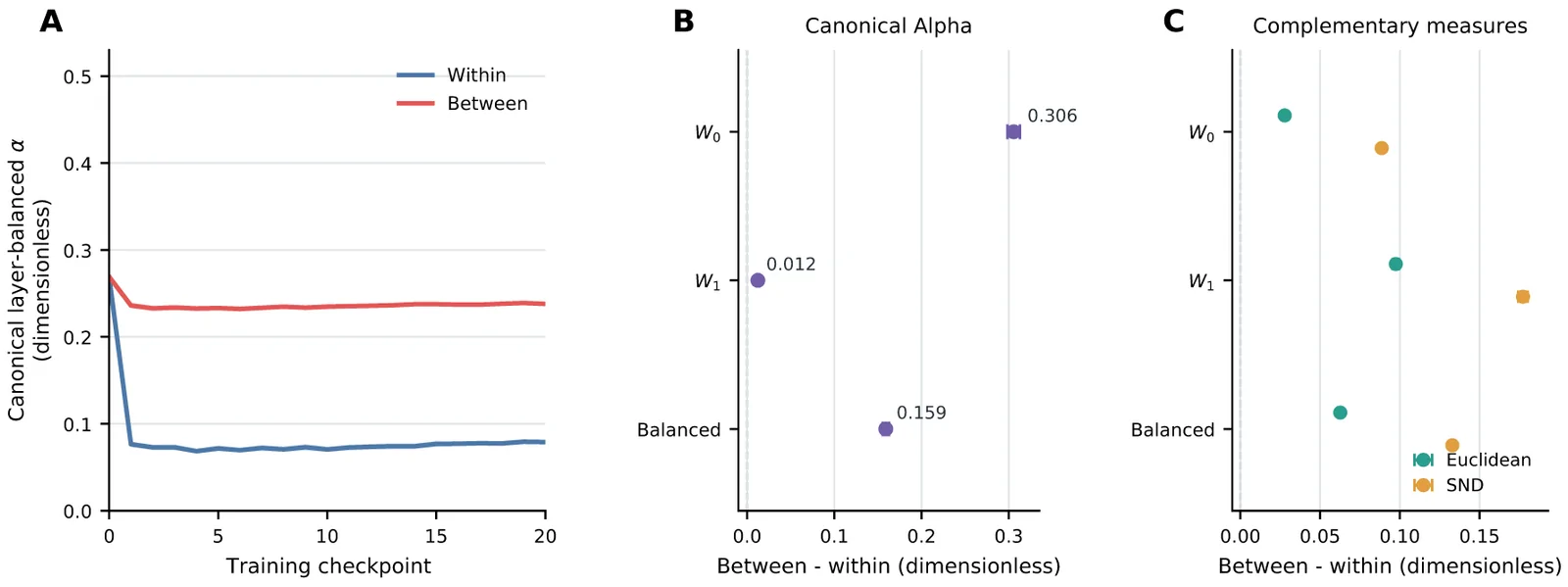
Dataset-dependent weight structure develops across both network layers. **(A)** Training trajectories of canonically gauged, layer-balanced Alpha dissimilarity (α = 2) for network pairs trained on the same dataset (within) or on different datasets (between). Lines show group estimates and shaded regions show 95% confidence intervals. **(B)** Between-minus-within Alpha contrasts at the final checkpoint for the input-to-hidden weights (*W*_0_), hidden-to-output weights (*W*_1_), and their layer-balanced mean. The Alpha panels use canonical weights and a shared direct canonical-Alpha profile Hungarian assignment based on incoming and outgoing profiles; the layer-balanced endpoint averages the two matrix values equally. The contrasts were 0.3057 (95% confidence interval, 0.2985–0.3129), 0.0123 (0.0113–0.0133), and 0.1590 (0.1553–0.1627), respectively. **(C)** Corresponding final contrasts for Euclidean dissimilarity and Structural Network Dissimilarity (SND). All contrasts were positive in both layers. Points show estimates and whiskers show 95% confidence intervals from a dataset-stratified delete-one-run jackknife across 30 independent training runs.

**Figure 3:**
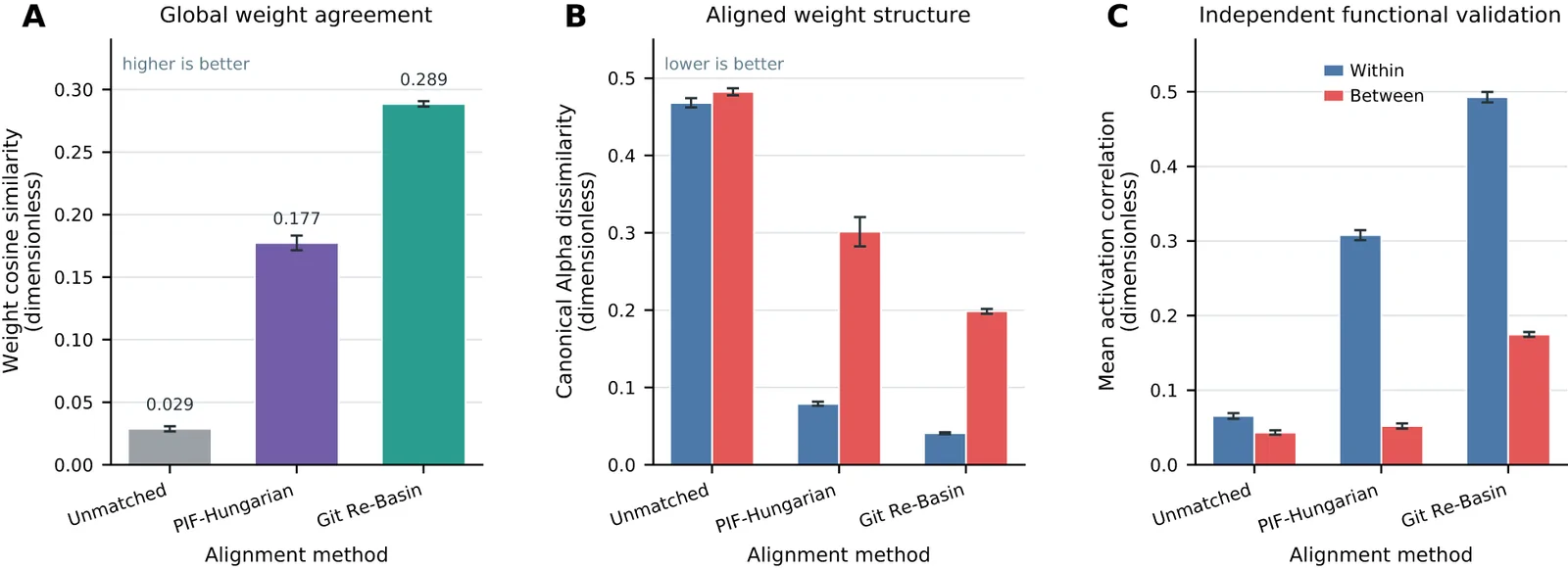
Git Re-Basin yields stronger neuron correspondence while PIF-Hungarian matching retains functional value. **(A)** Raw weight-cosine similarity at the final checkpoint, pooled over all 435 network pairs. Similarity increased from 0.0288 in the unmatched neuron order to 0.1774 after PIF-Hungarian matching and 0.2885 after Git Re-Basin. **(B)** Canonically gauged, layer-balanced Alpha dissimilarity separated into within-dataset and between-dataset pairs. The PIF-Hungarian label denotes the candidate-selected benchmark variant described in Section 2.6, with parameter-count-weighted candidate selection and layer-balanced canonical Alpha evaluation; it does not denote the direct assignment used in Figure 1. For PIF-Hungarian matching, the within and between estimates were 0.0789 and 0.3015; for Git Re-Basin, they were 0.0409 and 0.1985. Lower values indicate greater aligned weight similarity. **(C)** Independent functional validation by the mean correlation of matched hidden-neuron activations. In the unmatched, PIF-Hungarian, and Git-Re-Basin conditions, the correlations were, respectively 0.0655, 0.3078, and 0.4928 within datasets, and 0.0433, 0.0520, and 0.1747 between datasets. Bars show group estimates and whiskers show 95% confidence intervals obtained by a dataset-stratified delete-one-run jackknife across 30 complete training runs. Activation data were not used to infer the assignments. Both matching procedures therefore recovered functional correspondence beyond the original neuron numbering, but Git Re-Basin was consistently stronger.

### 2.7 Historical and methodological positioning of the proposed procedure

The PIF-Hungarian procedure originated in two Master's projects completed in our group in 2019. The dated thesis materials explicitly describe PIF-based Hungarian matching and weight-vector, multi-pass, and iterative Hungarian variants, but these materials were not archival research publications. The present paper provides the first formal and reproducible description of the procedure in a research article. This documented provenance does not establish priority for permutation-aware weight matching as a general idea, and we do not claim that the procedure is equivalent or superior to established methods. We treat it as an independently developed alignment variant and as a matching component designed for use with the Alpha diagnostic. Its performance was evaluated directly against Git Re-Basin, with both agreements and differences reported.

### 2.8 Git Re-Basin weight-matching baseline

We implemented the weight-matching objective of Git Re-Basin as described by Ainsworth et al. (2023). It seeks a hidden-unit permutation that maximizes the Frobenius inner product between corresponding weights of two networks. With one hidden layer, the similarity matrix is given by Equation 10:


$$\begin{array}{l} M=W_{0,A}^{T}W_{0,B}+W_{1,A}W_{1,B}^{T}. \end{array}$$ (10)

The permutation π maximizes $\underset{i}{\sum }M_{i,\pi \left(i\right)}$ and was obtained by applying the Hungarian algorithm to −*M*. In the present architecture there is only one permutation block, so this assignment is the global optimum of the Git Re-Basin weight-matching objective and requires neither coordinate-descent iterations nor random initialization.

Both the profile-based procedure and this one-hidden-layer Git Re-Basin implementation use a Hungarian solver. Their distinction lies in the assignment objective and parameter representation, not in the solver name.

For all 435 network pairs and all 21 checkpoints, we compared three states: the unaligned neuron order, Git Re-Basin alignment, and our canonically gauged Alpha alignment. The evaluation criteria were the raw total weight inner product and its cosine-normalized form, the raw weight root-mean-square error, layerwise and layer-balanced canonical Alpha, and matched-neuron activation correspondence. Algorithmic stability was tested on 30 predetermined final-checkpoint pairs. Network B was randomly renumbered 10 times per pair, the inferred assignment was composed back into a common reference indexing, and both the assignment and objective value were compared with the unperturbed solution. Applying any inferred permutation was also required to preserve logits numerically.

### 2.9 Weight-space dissimilarity measures

All local measures compare equally shaped arrays *A* and *B* containing *N* weights. The Euclidean baseline was the root-mean-square entrywise difference defined in Equation 11:


$$\begin{array}{l} d_{\text{Eucl}}\left(A,B\right)=\sqrt{\frac{1}{N}\underset{ij}{\sum }{\left(A_{ij}-B_{ij}\right)}^{2}}. \end{array}$$ (11)

SND compares weights relative to their sign and magnitude. For a pair *a* and *b* we used the definition in Equation 12:


$$\delta_{\text{SND}}(a,b)=\{\begin{array}{ll} 0, & a=b=0,\\ |\frac{a-b}{a+b}|, & \mathrm{sgn}(a)=\mathrm{sgn}(b)\neq 0,\\ 1, & \text{otherwise}, \end{array}$$ (12)

and $d_{\text{SND}}\left(A,B\right)=N^{-1}\underset{ij}{\sum }\delta_{\text{SND}}\left(A_{ij},B_{ij}\right)$. Denominators with absolute value at most 10^−12^ were assigned a contribution of one.

Alpha is an importance-weighted sign-disagreement dissimilarity. With exponent α>0, the importance and sign-disagreement terms were defined in Equation 13:


$$\begin{array}{l} p_{ij}=\frac{|A_{ij}B_{ij}{|}^{\alpha }}{\sum_{mn}|A_{mn}B_{mn}{|}^{\alpha }},\;s_{ij}=\text{1}\left[sgn\left(A_{ij}\right)\neq \text{sgn}\left(B_{ij}\right)\right], \end{array}$$ (13)

The resulting dissimilarity is given by Equation 14:


$$\begin{array}{l} d_{\alpha }\left(A,B\right)=\underset{ij}{\sum }p_{ij}s_{ij}. \end{array}$$ (14)

If the importance denominator was at most 10^−12^, the value was set to zero. We used α = 2 throughout. Alpha lies in [0, 1], is invariant to separate positive global rescalings of the two compared arrays, and emphasizes sign disagreements at weights that are large in both arrays. It is not a mathematical metric: in particular, it need not satisfy the triangle inequality and can be zero for different weight magnitudes with identical sign patterns. We therefore use the term *dissimilarity*.

After matching, every measure was computed separately for *W*_0_ and *W*_1_. For layerwise values *d*_0_ and *d*_1_, the layer-balanced summary was defined by Equation 15:


$$\begin{array}{l} D_{\text{bal}}=\frac{d_{0}+d_{1}}{2}, \end{array}$$ (15)

whereas the parameter-count-weighted summary was defined by Equation 16:


$$\begin{array}{l} D_{\text{size}}=\frac{|W_{0}|d_{0}+|W_{1}|d_{1}}{\left|W_{0}|+|W_{1}\right|}. \end{array}$$ (16)

Here |*W*_0_| = 50, 176 and |*W*_1_| = 640, so the size-weighted result assigns 98.7406% of its weight to *W*_0_. Layer-balanced values were used to prevent the input layer from numerically obscuring the output layer; size-weighted values were reported as a secondary aggregation.

### 2.10 Canonical ReLU gauge for Alpha

Positive homogeneity of ReLU creates a second function-preserving symmetry. For any hidden neuron *k* and *c*_*k*_>0, the transformation in Equation 17 leaves all logits unchanged:


$$\begin{array}{l} W_{0}\left[:,k\right]\mapsto c_{k}W_{0}\left[:,k\right],\;W_{1}\left[k,:\right]\mapsto W_{1}\left[k,:\right]/c_{k} \end{array}$$ (17)

leaves all logits unchanged. Raw Alpha is not invariant to this neuronwise transformation because it changes the relative importance weights within each matrix.

We therefore defined a canonical gauge separately for every nondegenerate hidden neuron. Let the two neuron norms be defined as in Equation 18:


$$\begin{array}{l} a_{k}=∥W_{0}\left[:,k\right]{∥}_{2},\;b_{k}=∥W_{1}\left[k,:\right]{∥}_{2}, \end{array}$$ (18)

and set $s_{k}=\sqrt{b_{k}/a_{k}}$. We transformed the weights as specified in Equation 19:


$$\begin{array}{l} W_{0}\left[:,k\right]\mapsto s_{k}W_{0}\left[:,k\right],\;W_{1}\left[k,:\right]\mapsto W_{1}\left[k,:\right]/s_{k}. \end{array}$$ (19)

Both neuron norms then equal $\sqrt{a_{k}b_{k}}$. This gauge is invariant to prior positive neuronwise rescaling and equivariant to neuron permutation. All neurons in the analyzed checkpoints had nonzero incoming and outgoing norms.

Gauge sensitivity was quantified at *E* = 20 for all 30 networks. For rescaling envelope *C*∈{1, 1.25, 1.5, 2, 4, 8}, we used $c_{k}=C^{z_{k}}$, where the 64 exponents were equally spaced from −1 to 1 and deterministically permuted separately for each network. Their geometric mean was one. Matching and raw Alpha calculation were repeated after every transformation. Function preservation was checked on 256 probe images by comparing logits and predicted classes, and canonicalized weights before and after rescaling were compared elementwise. Finally, canonical Alpha was recomputed for all 30 networks, all 21 checkpoints, and all 435 pairs.

### 2.11 Common probe set and hidden activations

Representation and matching-quality analyses used a fixed probe set constructed from the three test partitions. Using NumPy random seed 5,052,026, we sampled without replacement 100 images from every nominal class of every dataset, yielding 1,000 images per dataset and 3,000 in total. Class indices shared across datasets were used only for balancing and were not assumed to have common semantics. Each image was normalized once with the constants of its source dataset. The resulting 3, 000 × 784 single-precision matrix, its selected test indices, and a SHA-256 fingerprint were stored and reused unchanged for all networks.

For checkpoint *t* of network *r*, post-ReLU hidden activations were given by Equation 20:


$$\begin{array}{l} H_{r,t}=\mathrm{ReLU}\left(XW_{0,r,t}\right)\in {\mathbb{R}}^{3,000\times 64}. \end{array}$$ (20)

The output layer was not applied. Thus, every network received exactly the same numerical inputs, including images originating outside the dataset on which it had been trained.

### 2.12 Linear CKA

We compared hidden representations with linear CKA. Each activation matrix was centered over probe images, $\tilde{H}=H-\text{1}\bar{H}$, and the similarity between networks A and B was defined in Equation 21:


$$\begin{array}{l} \mathrm{CKA}\left(H_{A},H_{B}\right)=\frac{∥{\tilde{H}}_{A}^{T}{\tilde{H}}_{B}{∥}_{F}^{2}}{∥{\tilde{H}}_{A}^{T}{\tilde{H}}_{A}{∥}_{F}∥{\tilde{H}}_{B}^{T}{\tilde{H}}_{B}{∥}_{F}}. \end{array}$$ (21)

We report 1 − CKA so that larger values, as for the weight measures, indicate greater dissimilarity. Linear CKA is invariant to orthogonal transformations of the feature coordinates and therefore to hidden-unit permutations; it required no matching. Values were clipped to [0, 1] to remove numerical round-off. Main-cohort CKA was calculated for all 435 pairs at *E*∈{0, 1, 2, 5, 10, 20} and was compared with raw *W*_0_, *W*_1_, and layer-balanced Alpha by Pearson and Spearman correlations. These layer-specific Alpha evaluations use the raw-profile main-cohort matching described above; evaluating *W*_0_ alone should not be confused with inferring an assignment from incoming weights alone. Correlations were also evaluated separately within and between dataset groups to distinguish pairwise agreement from correlation induced by the group separation itself.

### 2.13 Matched-neuron activation correspondence

To evaluate an inferred permutation independently of its weight objective, we measured the correspondence of individual hidden-neuron activations. For every network and checkpoint, each column of *H* was centered across the 3,000 probe images and divided by its Euclidean norm. For a permutation π mapping network B onto network A, activation correspondence was defined in Equation 22:


$$\begin{array}{l} R_{\text{act}}\left(A,B;\pi \right)=\frac{1}{64}\overset{64}{\underset{k=1}{\sum }}\mathrm{corr}\left(H_{A}\left[:,k\right],H_{B}\left[:,\pi \left(k\right)\right]\right). \end{array}$$ (22)

This is the mean of 64 Pearson correlations and was computed for the unaligned neuron order, the canonical Alpha assignment, and the Git Re-Basin assignment at all 21 main checkpoints. Because activations were not used to infer either assignment, this measure provides an out-of-objective assessment of functional neuron correspondence. We did not use linear interpolation as a common endpoint because networks trained on different datasets do not share a unique training loss under which an interpolation barrier could be defined without an additional convention.

### 2.14 Task-vs.-dataset control

To separate target-rule effects from input-distribution effects, the task-control cohort comprised 20 networks trained on the same MNIST training and test images. Ten networks predicted digit identity with 10 logits; 10 predicted binary parity, *y*_parity_ = *y* mod 2, with two logits. Both tasks used the same normalization, hidden width, ReLU nonlinearity, zero fixed biases, optimizer, batch size, and 10-epoch schedule as the main cohort.

The experiment used 10 paired seed blocks, 401–410. Within each block, the identity and parity networks had bitwise identical initial *W*_0_ matrices and the same training-image order. Their output layers necessarily differed in shape. We therefore compared only *W*_0_ and its resulting hidden representation; *W*_1_ was not compared across tasks. For each weight measure, the 64 columns of *W*_0_ were assigned by Hungarian matching using distances between their 784-dimensional incoming weight vectors. Weight and CKA analyses used *E*∈{0, 1, 2, 5, 10, 20}.

For this control, the fixed representation probe comprised the first 200 test examples of each MNIST digit in IDX order, for 2,000 images in total. The two prespecified primary endpoints at *E* = 20 were the between-task minus within-task contrast for matched *W*_0_ Alpha with α = 2 and the analogous contrast for hidden 1 − CKA. Euclidean and SND contrasts and same-seed vs. different-seed cross-task pairs were secondary analyses. Because the output dimensions differed, the task-control *W*_0_ Alpha analysis was not subjected to the two-layer canonical gauge; CKA and the two additional weight measures provided complementary checks.

The whole-network Git Re-Basin objective implemented for the three-dataset cohort combines incoming and outgoing weight information and is not directly applicable to these cross-task pairs. The task-control inference therefore rests on incoming-weight Alpha-profile Hungarian assignment and hidden-representation comparisons, rather than on the whole-network benchmark.

### 2.15 High-resolution early-SGD experiment

The early-SGD cohort comprised five networks per dataset, 15 in total, with seeds 701–705 for MNIST, 801–805 for Fashion-MNIST, and 901–905 for KMNIST. Architecture, preprocessing, loss, SGD learning rate, momentum, and batch size were identical to the main experiment. To resolve the first optimization steps beyond the half-epoch sampling of the main cohort, checkpoints were stored after the optimizer-update counts listed in Equation 23:


$$\begin{array}{l} s\in \left\{0,1,2,4,8,16,32,48,64,96,128,160,192,235,352,470\right\} \end{array}$$ (23)

optimizer updates. With 235 mini-batches per epoch, this spans initialization through two full epochs.

At every checkpoint, Euclidean, SND, raw Alpha, canonical Alpha, and linear CKA were calculated for all 105 network pairs. Weight measures were evaluated by layer and by the layer-balanced average; CKA used the fixed 3,000-image probe set. For each measure, structural or representational onset was defined before calculation as the first post-initialization checkpoint at which the complete 95% confidence interval for the between-minus-within dataset contrast lay above zero.

Functional progress was defined separately for each dataset as in Equation 24:


$$\begin{array}{l} P_{d}\left(s\right)=\frac{A_{d}\left(s\right)-A_{d}\left(0\right)}{A_{d}\left(470\right)-A_{d}\left(0\right)}, \end{array}$$ (24)

where *A*_*d*_(*s*) is mean test accuracy over the five runs. The functional 90% time was the first checkpoint at which the mean of *P*_*d*_(*s*) across the three datasets reached at least 0.9. A calibration-then-consolidation sequence was to be supported only if this functional criterion preceded a clearly later canonical-Alpha onset in every dataset; simultaneous or earlier structural onset was treated as evidence against that sequence.

To quantify how far the parameters had moved while accuracy increased, we measured displacement from initialization separately for each layer ℓ using Equation 25:


$$\begin{array}{l} R_{\ell }\left(s\right)=\frac{∥W_{\ell }\left(s\right)-W_{\ell }\left(0\right){∥}_{F}}{∥W_{\ell }\left(0\right){∥}_{F}}. \end{array}$$ (25)

We additionally report the root-mean-square change per parameter defined in Equation 26:


$$\begin{array}{l} Q_{\ell }\left(s\right)=\frac{∥W_{\ell }\left(s\right)-W_{\ell }\left(0\right){∥}_{F}}{\sqrt{\left|W_{\ell }\right|}}. \end{array}$$ (26)

These descriptive displacement measures were summarized over the 15 independent runs by their mean and 95% Student-*t* confidence interval.

### 2.16 Statistical analysis

For the main cohort, the within-dataset estimate was the mean of all pairwise dissimilarities among different runs trained on the same dataset, pooled over the MNIST, Fashion-MNIST, and KMNIST groups. The between-dataset estimate was the mean over all MNIST–Fashion-MNIST, MNIST–KMNIST, and Fashion-MNIST–KMNIST pairs. The principal contrast was defined by Equation 27:


$$\begin{array}{l} \Delta ={\bar{D}}_{\text{between}}-{\bar{D}}_{\text{within}}, \end{array}$$ (27)

so positive values indicate dataset-dependent grouping. The same orientation was used in the task control. We report group estimates, contrasts, and 95% confidence intervals rather than treating the many dependent pairs as separate replicates.

Uncertainty in the main cohort was estimated with a dataset-stratified delete-one-run jackknife. Within each dataset stratum *g*, every one of the *n*_*g*_ = 10 complete runs was removed in turn, and the statistic was recomputed after removing all pair values incident to that run. If ${\hat{\theta }}_{g,-r}$ denotes such a replicate and ${\bar{\theta }}_{g}$ their mean, the variance estimate was given by Equation 28:


$$\begin{array}{l} \hat{\mathrm{Var}}\left(\hat{\theta }\right)=\underset{g}{\sum }\frac{n_{g}-1}{n_{g}}\overset{n_{g}}{\underset{r=1}{\sum }}{\left({\hat{\theta }}_{g,-r}-{\bar{\theta }}_{g}\right)}^{2}. \end{array}$$ (28)

Normal 95% intervals were formed as $\hat{\theta }\pm 1.95996\text{SE}$. The same run-level procedure was used for CKA contrasts, weight–CKA correlations, Git Re-Basin comparisons, and matched-neuron activation correspondence. Pair counts were reported descriptively but did not determine the inferential sample size.

For the identity-vs.-parity control, the independent unit was the paired seed block. Both networks belonging to one of the 10 blocks were deleted together, and the usual delete-one-block jackknife variance, $\left(B-1\right)B^{-1}\underset{b}{\sum }{\left({\hat{\theta }}_{-b}-\bar{\theta }\right)}^{2}$ with *B* = 10, was used. The densely sampled SGD experiment used the same dataset-stratified run-level jackknife as the main cohort, with five runs per dataset. Pearson and Spearman coefficients were computed over the relevant pair values, but their uncertainty was obtained by deleting whole runs. No network-pair-level null-hypothesis tests were used, and no claim of independence was based on the 435 pairwise observations.

### 2.17 Reproducibility and computational environment

All training trajectories were stored as compressed NumPy archives containing *W*_0_ and *W*_1_. Machine-readable manifests record labels, seeds, checkpoint indices, shapes, file paths, and SHA-256 hashes. Fixed probe matrices, selected source indices, analysis plans, long-format pair tables, jackknife replicates, summary files, validation reports, and figure-generation scripts were stored with each analysis. Distance matrices were checked for finiteness, symmetry, and zero diagonals. Independent direct recalculations were used to verify representative CKA, matching, and dissimilarity values. Hidden-unit permutations were checked to preserve the network function numerically, and canonicalization was checked to remove the tested positive ReLU rescalings.

Computations were run under Python 3.9.5 (Python Software Foundation, Beaverton, OR, USA) on 64-bit Windows (Microsoft Corporation, Redmond, WA, USA), using NumPy 1.23.5 (NumPy developers; https://numpy.org), SciPy 1.10.1 (SciPy developers; https://scipy.org), PyTorch 2.8.0+cpu (PyTorch community/PyTorch Foundation; https://pytorch.org), and Matplotlib 3.4.3 (Matplotlib development team; https://matplotlib.org) where plotting was required. Training and numerical analyses used an Intel x86-64 CPU with eight logical processors. Random seeds, deterministic probe selection, and per-epoch data-loader seeds were stored explicitly. The direct implementation of the one-hidden-layer Git Re-Basin objective avoided reliance on an external matching package. Together, the stored checkpoints and scripts reproduce every numerical endpoint reported in this study.

OpenAI Codex, powered by GPT-5 (OpenAI; accessed in 2026), assisted with language editing, organization of analysis code, and cross-document consistency checks. All AI-assisted code, numerical outputs, citations, and scientific interpretations were reviewed and verified by the authors, who take full responsibility for the work.

## 3 Results

### 3.1 Training was consistent across independent runs

Before asking whether training organized the weights in a reproducible way, we first established that the networks being compared had learned their respective classification problems reliably. As expected, test accuracy rose rapidly in all 30 independently initialized runs and then changed more gradually over the remaining epochs (Supplementary Figure S1). At the final checkpoint, mean accuracy was 97.37% for MNIST, 86.37% for Fashion-MNIST, and 85.99% for KMNIST. Run-to-run variation within each dataset was small, with standard deviations of 0.24, 0.47, and 0.70 percentage points, respectively, and no individual run failed to learn. The different performance levels across datasets were consistent with their different classification difficulty; the important control for the structural analyses was that independently initialized networks reached closely grouped performance within each dataset.

Test loss added one qualification to this otherwise uniform picture. For KMNIST, accuracy remained stable while loss rose again during later training (Supplementary Figure S1). This pattern is consistent with increasing confidence on the examples that remained misclassified, rather than with a loss of classification performance. The complete set of runs therefore provided reliable functional endpoints for comparison. Similar performance, however, could arise either because networks found related internal structures or because different structures supported the same behavior. We addressed this ambiguity by aligning the hidden neurons and comparing the resulting weights within and between datasets.

### 3.2 Dataset-dependent separation of aligned weight structure

Comparable performance within each dataset did not show whether the networks had learned related internal structures. To test this, we removed the positive ReLU scaling freedom, aligned the hidden neurons for each network pair, and measured canonically gauged, layer-balanced Alpha dissimilarity. The 435 network pairs were divided into 135 comparisons within the same dataset and 300 comparisons between different datasets (Figure 1A). At initialization, the two groups were indistinguishable: mean dissimilarity was 0.2688 within datasets and 0.2691 between datasets, giving a between-minus-within contrast of only 0.0003 with a confidence interval that included zero. Thus, the random initializations contained no detectable dataset grouping before learning began.

Training changed this relation quickly and persistently. After the first half epoch, mean dissimilarity had fallen to 0.0765 within datasets but remained at 0.2361 between datasets, producing a contrast of 0.1596 (Figure 2A). The separation then remained stable across the sampled trajectory. At the final checkpoint, within-dataset dissimilarity was 0.0789 and between-dataset dissimilarity was 0.2379, approximately three times as large (Figure 1B). Their difference was 0.1590 (95% confidence interval, 0.1553–0.1627). Unlike the similar performance reached within each dataset, this structural grouping was not guaranteed by the training objective: independently initialized networks could have solved the same problem through unrelated aligned weights. Instead, training placed networks from the same dataset in more closely related regions of aligned weight space. The non-zero within-dataset dissimilarity also shows that they did not approach one common parameter point. Because the primary measure averaged the two weight matrices, however, it could not show whether both layers contributed to the separation or whether one layer carried most of it. We therefore resolved the effect by layer.

### 3.3 Layer-resolved results

Resolving the layer-balanced result revealed a pronounced asymmetry between the two weight matrices (Figure 2B). For the input-to-hidden weights *W*_0_, mean canonical Alpha dissimilarity was 0.1511 within datasets and 0.4568 between datasets. The resulting contrast was 0.3057 (95% confidence interval, 0.2985–0.3129). The hidden-to-output weights *W*_1_ showed much smaller absolute values, but the same ordering: dissimilarity was 0.0067 within datasets and 0.0190 between datasets, giving a positive contrast of 0.0123 (0.0113–0.0133). Thus, both layers separated networks by dataset, although *W*_0_ contributed most of the absolute canonical-Alpha effect.

This dominance was not a general property of every weight comparison. With metric-specific matching, the final between-minus-within Euclidean contrasts were 0.0279 for *W*_0_ and 0.0975 for *W*_1_, while the corresponding SND contrasts were 0.0886 and 0.1772 (Figure 2C). All four confidence intervals lay entirely above zero. The layer-balanced and parameter-count-weighted summaries were likewise positive across Alpha, Euclidean dissimilarity, and SND (Supplementary Figure S2). The consistent direction across layers and measures was expected if the dataset influenced the network as a whole; the marked shift in which layer carried the larger numerical contrast shows that effect magnitudes cannot be compared across measures as though they described the same geometry. Most importantly, no single layer or distance definition was solely responsible for the dataset grouping. A group-level effect, however, could still be driven by a small subset of unusual training runs. We therefore examined how consistently individual networks contributed to the separation and estimated uncertainty by removing complete runs.

### 3.4 Run-level robustness and uncertainty

The agreement across layers and measures still left open whether a few unusual networks dominated the group averages. We therefore assigned each run a descriptive contribution: its mean distance to networks trained on other datasets minus its mean distance to networks trained on the same dataset. Every one of the 30 runs contributed positively to the final separation for Alpha in both layers. The same direction held across Euclidean dissimilarity, SND, the layer-balanced summaries, and the parameter-count-weighted summaries (Supplementary Figure S3). The effect was therefore distributed across all three datasets and did not depend on a small set of extreme runs.

Because one network participates in many pairwise comparisons, the 435 pair values are not independent replicates. We therefore estimated all confidence intervals by deleting one complete run at a time within its dataset stratum. Under this conservative run-level analysis, the primary canonical-Alpha contrast remained 0.1590, with its 95% confidence interval bounded well above zero (0.1553–0.1627). This established that the dataset grouping was reproducible across initializations. It did not yet establish what aspect of the training condition produced the grouping, because MNIST, Fashion-MNIST, and KMNIST differ in both their images and their classification targets. To separate these influences, we next changed the objective while keeping the input images fixed.

### 3.5 Different objectives induce different structures on identical images

The task-control experiment paired two networks within each seed block. Both received the same MNIST images in the same order and began with exactly the same *W*_0_, but one predicted digit identity and the other predicted parity. As expected, both objectives were learned reliably, reaching mean final accuracies of 97.25 and 98.60%, respectively. At initialization, every same-seed cross-task pair was identical in *W*_0_ and in its hidden representation. The negative pooled task contrast at this point was therefore a consequence of the paired design: the cross-task group contained identical same-seed pairs, whereas the within-task group compared different seeds.

Training reversed this initial relation (Figure 4). At the final checkpoint, matched *W*_0_ Alpha dissimilarity was 0.0367 within tasks and 0.0812 between tasks, giving a between-task-minus-within-task contrast of 0.0445 (95% confidence interval, 0.0411–0.0479). Hidden 1 − CKA was likewise lower within tasks than between them (0.1969 vs. 0.3335), with a contrast of 0.1366 (0.1244–0.1487). Even the especially stringent same-seed cross-task pairs remained clearly separated: their mean Alpha dissimilarity was 0.0786, close to the value of 0.0815 for cross-task pairs with different seeds. Euclidean and SND comparisons gave the same qualitative result (Supplementary Figure S4). Thus, changing only the learned rule redirected both the weights and the hidden representation. The main dataset effect could not be attributed to the input distribution alone.

**Figure 4:**
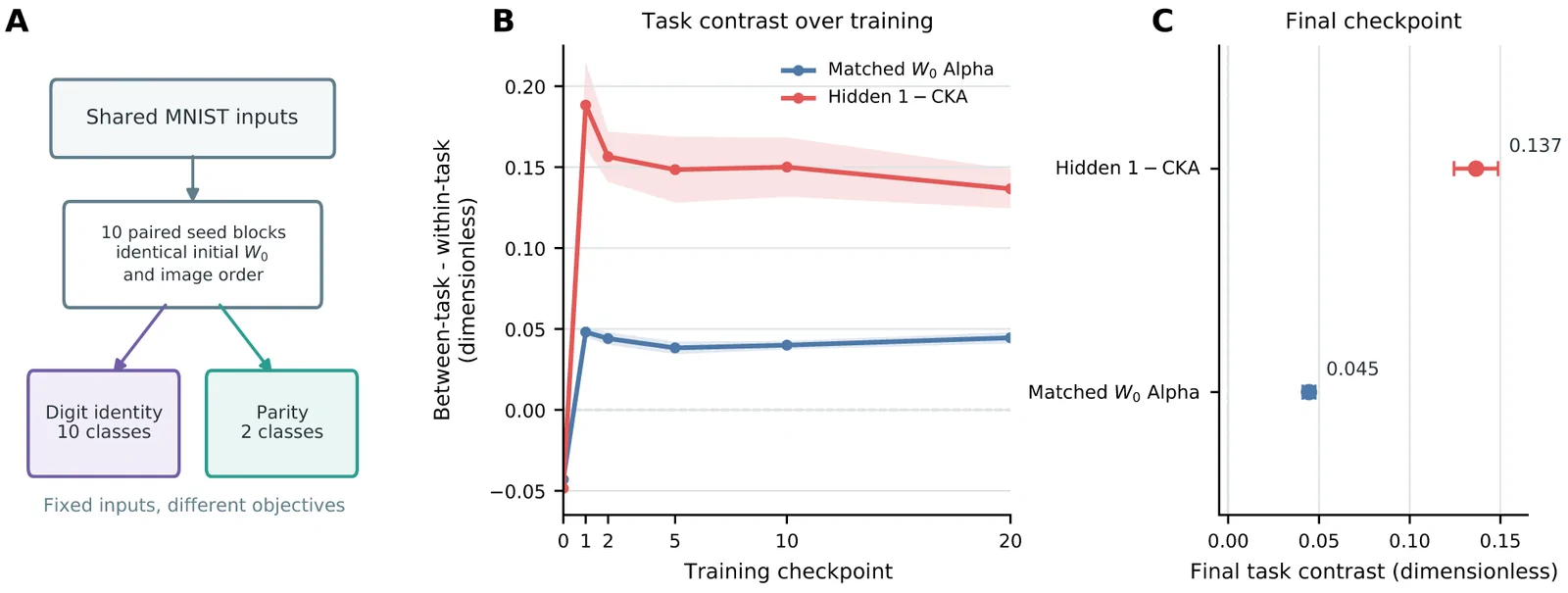
Changing the objective separates networks trained on identical images. **(A)** Ten paired seed blocks were trained on the same MNIST images with identical input-to-hidden initialization and image order, but with either 10-class digit identity or binary parity as the prediction objective. Because the output dimensions differed, weight comparisons were restricted to *W*_0_. Alpha is raw, without the two-layer canonical gauge, and uses incoming-weight Alpha-profile Hungarian assignment. The implemented whole-network Git Re-Basin objective is not directly applicable across these tasks. **(B)** Between-task-minus-within-task contrasts over training for permutation-matched *W*_0_ Alpha dissimilarity (α = 2) and hidden-representation dissimilarity (1 − CKA). Lines show estimates and shaded regions show 95% confidence intervals. The negative pooled contrasts at initialization are an expected consequence of the paired design: same-seed cross-task networks had identical initial *W*_0_, whereas within-task pairs joined different seeds. **(C)** At the final checkpoint, the task contrast was 0.0445 (95% confidence interval, 0.0411–0.0479) for matched *W*_0_ Alpha and 0.1366 (0.1244–0.1487) for hidden 1 − CKA. Points show estimates and whiskers show 95% confidence intervals obtained by deleting one complete paired seed block at a time.

### 3.6 Hidden representations confirm the dataset-dependent separation

The task control linked weight structure to hidden activity, but the main three-dataset result could still have reflected properties specific to Alpha and neuron matching. We therefore compared hidden activation spaces with linear CKA, which does not require a one-to-one neuron assignment. At initialization, neither matched *W*_0_ Alpha nor hidden 1 − CKA separated within- from between-dataset pairs. Once training began, both measures developed the same broad organization (Figure 5A).

**Figure 5:**
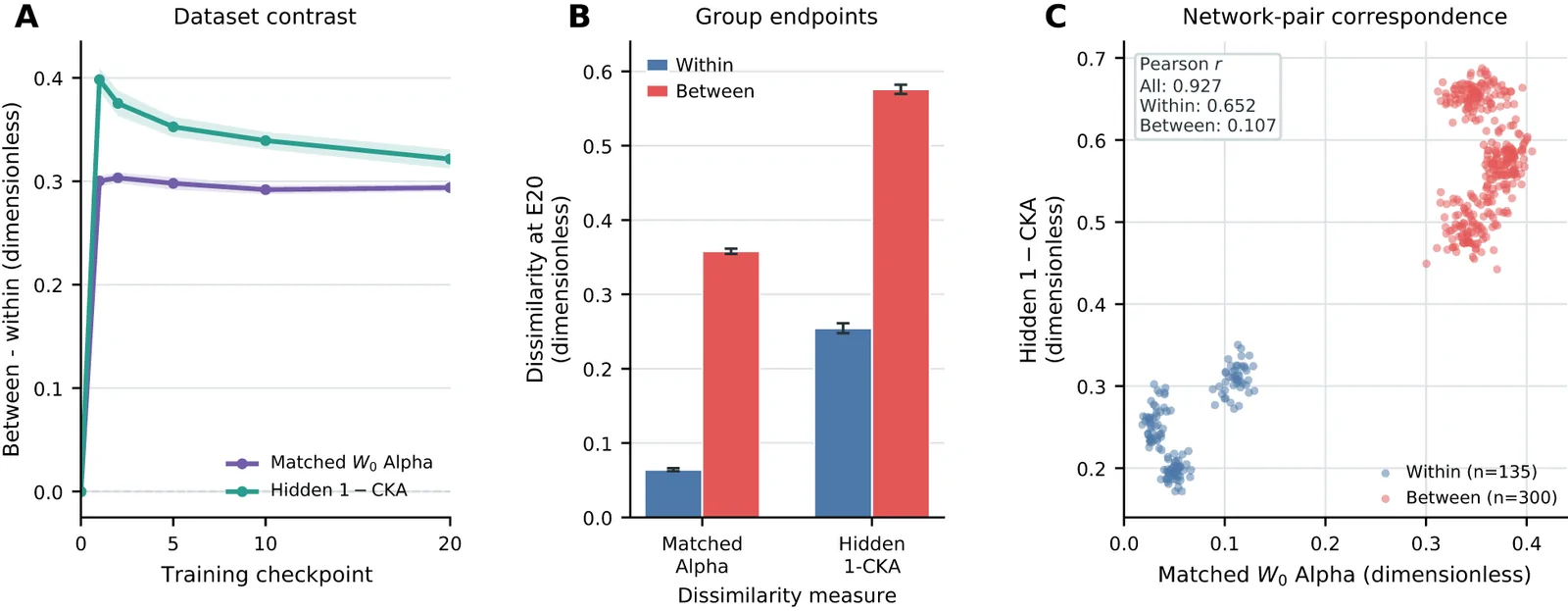
Representation-space similarity independently confirms weight-space clustering. **(A)** Between-dataset-minus-within-dataset contrasts across six training checkpoints for permutation-matched *W*_0_ Alpha dissimilarity (α = 2) and hidden-representation dissimilarity (1 − CKA). Lines show estimates and shaded regions show 95% confidence intervals. Both contrasts were compatible with zero at initialization and positive at every sampled checkpoint after training began. Alpha evaluates raw *W*_0_ after the main-cohort PIF-initialized Alpha-profile Hungarian procedure, which uses both incoming and outgoing profiles; no layer aggregation or canonical gauge is used for this *W*_0_ endpoint. **(B)** Separate group estimates at the final checkpoint. Matched *W*_0_ Alpha was 0.0641 within datasets and 0.3579 between datasets; hidden 1 − CKA was 0.2544 and 0.5758, respectively. Error bars show 95% confidence intervals. **(C)** Pairwise relationship between matched *W*_0_ Alpha and hidden 1 − CKA for all 435 network pairs at the final checkpoint. The overall Pearson correlation was 0.9269 (95% confidence interval, 0.9162–0.9377), but it was 0.6524 (0.5268–0.7780) within datasets and 0.1071 (−0.0823–0.2965) between datasets. Thus, the high pooled correlation primarily reflects the common group separation and does not imply interchangeable pairwise information. Confidence intervals were obtained by a dataset-stratified delete-one-run jackknife across 30 independent networks; pair counts are descriptive.

At the final checkpoint, hidden 1 − CKA was 0.2544 within datasets and 0.5758 between datasets (Figure 5B). The resulting contrast of 0.3215 had a 95% confidence interval of 0.3123–0.3306. For the corresponding matched raw *W*_0_ Alpha comparison, within- and between-dataset dissimilarities were 0.0641 and 0.3579, giving a contrast of 0.2938 (0.2901–0.2975). It was reassuring rather than surprising that two measurements of different objects recovered the same group structure: networks trained on the same dataset had both more similar aligned input weights and more similar hidden representations. Yet agreement at the group level does not imply that the two measures rank every network pair in the same way. We examined that finer relation next.

### 3.7 Alpha and CKA agree on groups but differ in pairwise detail

Across all 435 pairs, matched *W*_0_ Alpha and hidden 1 − CKA were strongly correlated at the final checkpoint (Pearson *r* = 0.9269, 95% confidence interval, 0.9162–0.9377; Figure 5C). Taken alone, this high value could suggest that the two measures carried nearly interchangeable information. However, the scatter contained two distinct clouds corresponding to within- and between-dataset comparisons. Their separation inflated the pooled correlation.

When the groups were examined separately, the association was more limited. It remained moderately positive among within-dataset pairs (*r* = 0.6524, 0.5268–0.7780), but fell to *r* = 0.1071 among between-dataset pairs, whose confidence interval included zero (−0.0823–0.2965). Spearman correlations led to the same conclusion (Supplementary Figure S5). Alpha and CKA therefore agreed strongly about which pairs belonged to the same broad dataset-defined group, while preserving different fine-grained information within those groups. This distinction made it important to test a separate limitation of the weight measure: Alpha might change when functionally equivalent ReLU networks are written at different neuronwise scales.

### 3.8 Alpha is gauge-sensitive but the principal result is robust

Positive rescaling of a ReLU neuron can multiply its incoming weights and divide its outgoing weights without changing any network output. We used this symmetry to stress-test raw Alpha over rescaling envelopes from *C* = 1 to *C* = 8. Predictions remained identical in every check and the largest absolute logit difference was below 5 × 10^−14^. Raw Alpha nevertheless changed materially (Supplementary Figure S6). At the strongest rescaling, the layer-balanced dataset contrast fell from 0.1559 to 0.1008, about 65% of its original size, although its confidence interval remained entirely positive (0.0947–0.1070). The raw numerical effect size is therefore not invariant to this function-preserving transformation.

To remove that freedom, we rescaled each hidden neuron to a canonical gauge in which its incoming and outgoing weight norms were equal. This operation recovered identical canonical weights from every stress-tested version to numerical precision. When all 9,135 pair-checkpoint comparisons were recalculated in this gauge, the final layer-balanced contrast was 0.1590 (0.1553–0.1627), close to the original raw value and robust across the full trajectory. Canonicalization therefore preserved the principal dataset separation while exposing why raw Alpha values should not be treated as intrinsic properties of a network function. Having established the endpoint across runs, measures, representations, tasks, and gauges, we then asked when the separation first emerged during learning.

### 3.9 Dataset-specific structure emerges during the first SGD updates

The half-epoch checkpoints showed that dataset grouping appeared early, but they could not resolve the first optimization steps. We therefore trained a separate densely sampled set of five networks per dataset and recorded the first two epochs. The earliest measurements revealed a striking imbalance between functional progress and global movement in parameter space (Figures 6A, B). Mean test accuracy across datasets rose from 0.1043 at initialization to 0.4146 after only two SGD updates; MNIST alone reached 0.5105. At the same point, the mean relative Frobenius displacement from initialization was only 0.0815 in *W*_0_ and 0.0588 in *W*_1_, corresponding to root-mean-square changes per parameter of 0.00168 and 0.00423. Substantial functional improvement therefore accompanied modest overall weight movement.

**Figure 6:**
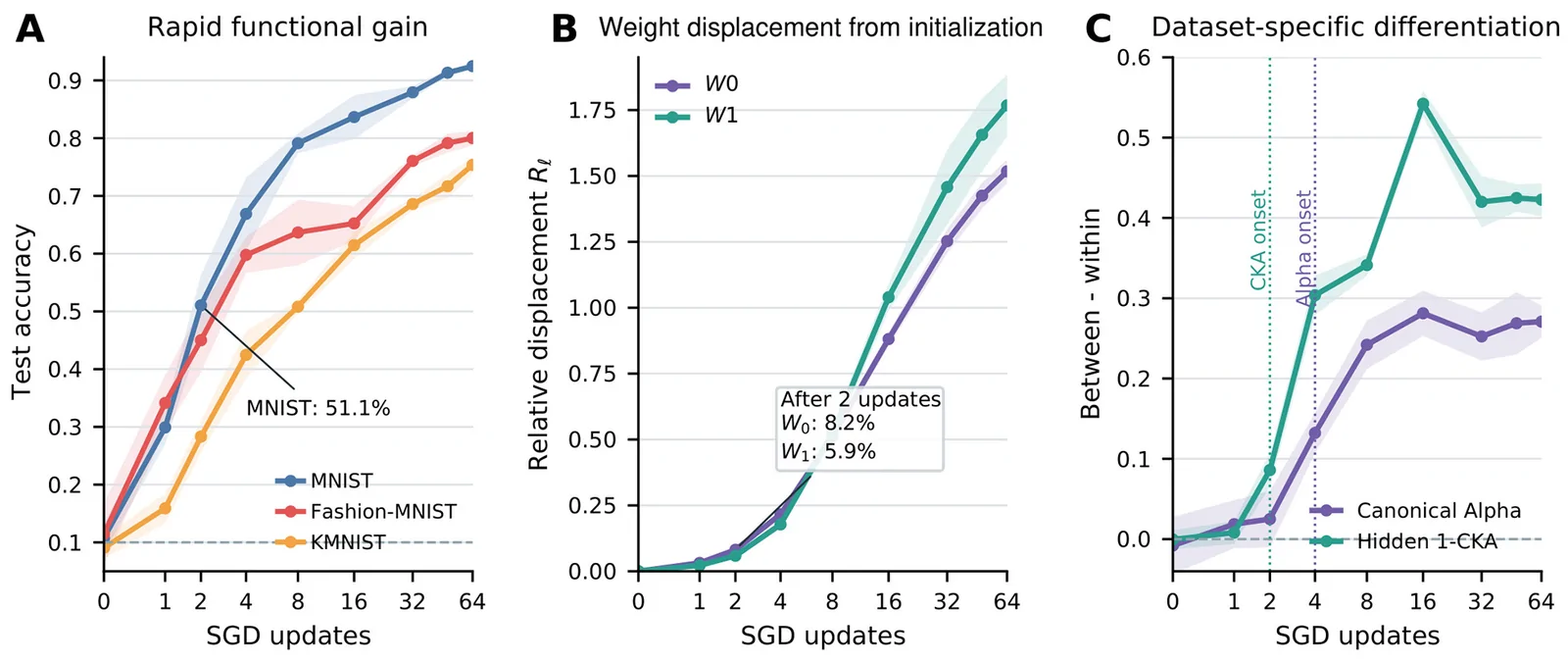
Small early weight changes accompany rapid functional gains and dataset-specific differentiation. **(A)** Test accuracy during the first 64 SGD updates for MNIST, Fashion-MNIST, and KMNIST. Lines show means over five independent runs per dataset and shaded regions show 95% Student-*t* confidence intervals; the dashed line marks chance accuracy. After two updates, mean accuracy across all three datasets was 0.4146, and MNIST accuracy was 0.5105. **(B)** Relative Frobenius displacement from initialization for the input-to-hidden (*W*_0_) and hidden-to-output (*W*_1_) layers, averaged over all 15 runs. After two updates, the displacements were 0.0815 and 0.0588, respectively; the corresponding root-mean-square changes per parameter were 0.00168 and 0.00423. Shaded regions show 95% Student-*t* confidence intervals. **(C)** Between-minus-within dataset contrasts for canonically gauged, layer-balanced Alpha dissimilarity and hidden 1 − CKA. Lines show estimates and shaded regions show 95% confidence intervals from a dataset-stratified delete-one-run jackknife. The first confidence interval entirely above zero occurred at two updates for CKA and four updates for canonical Alpha, whereas the prespecified 90% point of early accuracy progress was reached at 64 updates. During the first few updates, rapid functional learning accompanied modest global parameter displacement and already overlapped with dataset-specific structural and representational differentiation; this proximity to initialization does not describe the full interval shown.

These small changes were already organized by dataset (Figure 6C). Hidden 1 − CKA produced the first confidence interval entirely above zero after two updates, with a between-minus-within contrast of 0.0860 (0.0655–0.1066). Canonical Alpha crossed the same prespecified threshold after four updates, when its contrast was 0.1322 (0.1072–0.1571). Raw Alpha was detectable after two updates, while Euclidean dissimilarity and SND became detectable after four (Supplementary Figure S7). More unexpectedly than the rapid rise in accuracy itself, dataset-specific representational and weight structure was therefore measurable while the networks still remained close to their random starting points.

### 3.10 No evidence for a calibration-then-consolidation sequence

The early analysis was designed to distinguish two possible temporal accounts. Networks might first calibrate their function and only later consolidate a dataset-specific internal structure, or functional and structural differentiation might develop together. We defined functional calibration in advance as the point at which the mean trajectory had completed at least 90% of its accuracy gain over the observed early-training window. This threshold was reached after 64 updates across datasets, and after 32, 48, and 96 updates for MNIST, Fashion-MNIST, and KMNIST separately.

Representation-space separation after two updates and canonical weight-space separation after four therefore preceded, rather than followed, the functional threshold. The data do not support a calibration-then-consolidation sequence in which most functional learning is complete before dataset-specific structure begins. Instead, structural and representational differentiation overlapped with the rapid initial gain in accuracy. This negative result rules out the proposed two-stage timing in the present setting, although it does not by itself identify the dynamical mechanism behind the small early updates. Because all weight results still required a choice of neuron assignment, the final remaining question was whether they depended on the particular matching procedure.

### 3.11 Git Re-Basin provides the strongest neuron alignment

We compared PIF-Hungarian matching with Git Re-Basin and with the original, unmatched neuron order. At the final checkpoint, pooled raw weight-cosine similarity rose from 0.0288 without matching to 0.1774 after PIF-Hungarian matching and 0.2885 after Git Re-Basin (Figure 3A). Git Re-Basin was expected to perform well on this quantity because its objective directly increases the weight inner product. Its advantage, however, also transferred to a criterion it did not optimize directly. Pooled canonical, layer-balanced Alpha dissimilarity was 0.4780 without matching, 0.2324 after PIF-Hungarian matching, and 0.1496 after Git Re-Basin. The Git-Re-Basin advantage over PIF-Hungarian matching was 0.0828 (95% confidence interval, 0.0703–0.0953).

Both methods preserved the dataset-dependent ordering, but Git Re-Basin produced lower canonical Alpha dissimilarity both within and between datasets (Figure 3B). The two assignments agreed on only 16.5% of hidden neurons on average, showing that they found substantially different correspondences. Their relative ordering remained stable across training and both were invariant to random renumbering of the input networks (Supplementary Figure S8). Git Re-Basin was therefore the stronger weight-based alignment method in this architecture. This result did not make the older procedure functionally meaningless, however; that required an evaluation independent of both weight objectives.

### 3.12 Activation correspondence independently validates PIF-Hungarian matching

We evaluated each assignment by the correlation between matched hidden-neuron activations, using probe responses that had not entered either matching objective. Pooled mean activation correlation was 0.0502 in the unmatched order, 0.1314 after PIF-Hungarian matching, and 0.2734 after Git Re-Basin (Figure 3C). PIF-Hungarian matching therefore improved on the arbitrary neuron numbering by 0.0812 (95% confidence interval, 0.0780–0.0844). The improvement was especially clear within datasets: mean correlation rose from 0.0655 to 0.3078, and every one of the 135 within-dataset pairs improved (Supplementary Figure S9).

The limitation appeared in the harder between-dataset comparisons. There, PIF-Hungarian matching increased mean correlation only from 0.0433 to 0.0520 and improved 69.7% of pairs. Git Re-Basin reached correlations of 0.4928 within datasets and 0.1747 between datasets, exceeded PIF-Hungarian matching for all 435 pairs, and held an overall advantage of 0.1421 (0.1380–0.1461). Thus, the independently developed PIF-Hungarian procedure recovered genuine functional correspondence, particularly among networks trained on the same dataset, but it did not match the performance of Git Re-Basin. Together, the Results support task-dependent clustering in aligned weights while distinguishing that empirical conclusion from any claim that one alignment is uniquely correct or that the networks implement an identical algorithm.

## 4 Discussion

### 4.1 Principal findings

Independently initialized neural networks need not organize their internal parameters in the same way, even when they solve the same problem equally well. Here, however, training repeatedly produced dataset- and task-dependent structure after hidden-neuron permutations and positive ReLU rescalings had been taken into account. Networks trained on the same dataset were closer in aligned weight space than networks trained on different datasets, and their hidden representations showed the same broad grouping. The effect occurred in both weight matrices, survived run-level uncertainty estimates and several dissimilarity measures, and remained visible after the objective was changed while the input images and initial hidden weights were held fixed. The central result is therefore not merely that the networks learned reliably, but that the training condition left a reproducible signature in both parameters and representations.

Two results sharpen this conclusion. First, the signature appeared within the first few SGD updates, while the weights had moved only modestly from their random initial values and accuracy was already rising strongly. Structural differentiation therefore overlapped with functional learning instead of following it as a separate consolidation phase. Second, the established Git Re-Basin procedure aligned the networks more effectively than our PIF-Hungarian procedure. This limits the methodological claim we can make for the latter, but it also shows that the dataset effect did not depend on one favorable matching rule; the task effect was assessed separately with incoming-weight matching and CKA. These findings support task-dependent clustering in symmetry-adjusted network space. They do not, by themselves, show that independently trained networks implement an identical algorithm, approach a single parameter point, follow attractor dynamics, or undergo an evolutionary process.

### 4.2 What the within-/between separation means

The lower within-dataset than between-dataset dissimilarity is best understood as a repeatable structural signature of the learning problem. The absence of this grouping at initialization shows that it was not already present in the sampled random weights. Its appearance during training then shows that shared constraints—the images, labels, architecture, objective, and optimizer—biased otherwise independent trajectories toward more closely related regions. The non-zero distances within every dataset are equally important: the networks did not converge to one common weight vector. “Clustering” therefore describes the result more accurately than convergence to a point.

This interpretation also separates three levels that can otherwise be confused. Parameter similarity asks whether symmetry-adjusted weights resemble one another. Representation similarity asks whether populations of hidden units organize responses to a common probe set in related ways. Algorithmic similarity would make the stronger claim that the networks use the same sequence of computations or causal strategy. Our weight and CKA results address the first two levels. Their agreement makes a purely numerical matching artifact unlikely, but neither measure identifies a unique computation, and many parameterizations or representations can support the same input–output behavior. The present data therefore provide evidence for related internal organization, not for identity of learned algorithms.

### 4.3 Dataset effects vs. task effects

The strong separation among MNIST, Fashion-MNIST, and KMNIST could initially have had two explanations. Networks may respond differently because the image distributions differ, or because the target rules demand different decisions. The especially large Alpha contrast in the input-to-hidden weights makes a contribution from image statistics unsurprising: these weights directly encounter the pixels. Yet each dataset also defined its own classification problem, so the main cohort alone could not distinguish input from objective.

The identity-vs.-parity control resolved part of this ambiguity. Networks saw the same MNIST images in the same order and began from identical hidden weights within each seed pair, but learned different target rules. Training nevertheless separated both their incoming weights and hidden representations by task. Thus, the input distribution alone cannot explain the main result; the objective can redirect internal structure even when the sensory evidence is fixed. At the same time, the control does not assign a precise fraction of the main-cohort effect to images and labels. Identity and parity differ in output dimensionality, which also prevented a direct cross-task comparison of the output layer. The warranted conclusion is therefore that both the input distribution and the learned rule can shape internal organization, rather than that either source has been isolated completely.

### 4.4 Structural differentiation overlaps with functional learning

The most visually striking early result was that accuracy improved substantially after only a few SGD updates although the global weight displacement was still small. Small movement in a high-dimensional parameter vector can nevertheless cause coordinated changes in many activations, so rapid learning near initialization is not paradoxical. What was less expected was that these early changes already carried a reliable dataset signature: representation-space separation was detectable after two updates and canonical weight-space separation after four.

This timing rules out the specific calibration-then-consolidation account tested here. Under that account, the networks would first acquire most of their performance and only later settle into dataset-specific internal structure. Instead, weight, representation, and accuracy trajectories began to differentiate on overlapping timescales, long before the prespecified functional threshold was reached. This negative result matters because an appealing two-stage narrative could otherwise be read into coarsely sampled checkpoints.

Rejecting a sequential calibration-then-consolidation account leaves open a structural explanation for the effectiveness of the earliest small updates. We call this the target-rich landscape hypothesis: hidden-neuron permutations generate many equivalent realizations of a functional solution, and positive ReLU rescalings extend these symmetry orbits continuously. Flat directions and additional nearly equivalent solutions could further enlarge the useful regions, although they are not necessarily exact functional symmetries. A random initialization need not be close to one selected canonical optimum if it is close to some task-compatible solution set under an appropriately defined symmetry-adjusted distance. Crucially, equivalent copies collapse to one class in a quotient space, so symmetry multiplicity alone does not prove a high density of distinct useful classes or proximity to them. The mechanistic hypothesis is that the geometry and accessibility of these sets allow small coordinated updates to generate rapid functional gains and concurrent dataset-dependent differentiation. We did not measure initial distances to solution classes or map basin density, so the present observations motivate rather than establish this account.

The small early displacement also invites comparison with neural-tangent-kernel and lazy-training regimes, in which a network can change its function while remaining close to its initialization (Jacot et al., 2018; Chizat et al., 2019). Our observation is compatible with that broad possibility, but it does not establish it. We did not measure kernel stability, compare the trained network with its linearization, or vary the parameterization and width in the way required to diagnose lazy training. The safer conclusion is descriptive: in these finite, shallow networks, a modest but coordinated parameter change was sufficient for rapid functional progress and dataset-dependent internal differentiation.

### 4.5 Interpretation and limitations of Alpha

Alpha helped expose this organization because it asks a specific question: do corresponding parameters disagree in sign, and are those disagreements concentrated among weights that are large in both networks? This importance-weighted sign-disagreement view is intuitive for comparing aligned networks, but it should not be mistaken for a general metric. Two weight arrays with identical signs but different magnitudes have Alpha dissimilarity zero, so the identity-of-indiscernibles property fails; metric triangle properties have not been established either. Alpha is therefore a dissimilarity tailored to one aspect of weight structure.

The ReLU rescaling experiment revealed a second limitation. Raw Alpha changed when incoming and outgoing weights were rescaled in a way that preserved the network function. Placing every hidden neuron in a canonical norm gauge removed the tested scaling freedom and preserved the main dataset contrast. We therefore regard canonical Alpha as the preferred form for this architecture. Canonicalization is still a representational convention, not a quotient over every possible functional equivalence, and it does not turn Alpha into a metric.

No single measure should carry the full interpretation. Euclidean dissimilarity and SND confirmed the direction of the group effect, but assigned different relative magnitudes to the two layers. CKA recovered the same broad grouping without neuron matching, yet agreed much less with Alpha on the ranking of individual between-dataset pairs. These differences are informative: they show that the robust result is the group separation, whereas statements about exactly how far two networks are apart remain measure-dependent.

### 4.6 Contribution of the PIF-Hungarian procedure

The PIF-Hungarian procedure was developed in two Master's projects completed in our group in 2019, but it was not archivally published at that time. Its value in the present study is that the method is now defined completely enough to reproduce: it combines PIF ordering, neuron profiles, and a Hungarian assignment under an explicit dissimilarity. The activation analysis provides an external validation of that assignment. PIF-Hungarian matching improved mean correspondence between matched neuron responses, and it improved every within-dataset pair relative to the arbitrary original numbering. It therefore recovered genuine functional structure rather than merely optimizing a weight statistic.

That positive result does not imply priority for permutation-aware matching, general novelty of Hungarian alignment, or state-of-the-art performance. Related matching ideas are established in the literature, and the dated 2019 thesis records document provenance within our group rather than priority over the broader literature. Moreover, the present implementation was evaluated only for one hidden layer; the proposed alternating extension to deeper networks was not tested here. We therefore present PIF-Hungarian matching as a historically independent and now reproducibly documented variant whose behavior can be evaluated alongside stronger baselines.

### 4.7 Lessons from the Git Re-Basin comparison

Git Re-Basin produced the strongest alignment by every criterion used in the direct comparison (Ainsworth et al., 2023). Its advantage in pooled weight-cosine similarity was expected because that quantity is closely related to its matching objective. More importantly, the advantage transferred to canonical Alpha and to matched-neuron activation correlations, neither of which was directly optimized. Git Re-Basin also selected substantially different neuron correspondences from PIF-Hungarian matching. For this architecture, there is therefore no basis for presenting our procedure as the preferred optimizer of neuron assignments.

The canonical-Alpha/Hungarian analysis remains the primary diagnostic because its longitudinal behavior is an explicit object of the study. Git Re-Basin serves as an external benchmark for assessing the dependence of the observed dataset separation on the alignment procedure. Its stronger performance defines an important limitation of our method, while the preservation of the within-vs.-between dataset ordering supports the substantive group-level result. Future studies should select their primary alignment according to their scientific objective and include strong external baselines; the present comparison does not require replacing the diagnostic framework being evaluated. This whole-network comparison does not establish alignment-method robustness for the identity-vs.-parity control. Even then, a better assignment should not be confused with a uniquely correct biological or computational correspondence between neurons; it is the optimum of a stated comparison objective.

### 4.8 Relation to prior work

Our findings extend rather than originate the idea that independently trained networks can develop related internal structure. Convergent-learning studies compared features learned by separate networks (Li et al., 2015), while SVCCA, projection-weighted CCA, CKA, and related methods provided increasingly careful ways to compare representations across models (Raghu et al., 2017; Morcos et al., 2018; Kornblith et al., 2019; Csiszárik et al., 2021). Work on permutation symmetry and model merging has likewise shown why raw neuron order is not an appropriate coordinate system for parameter comparison (Entezari et al., 2022; Ainsworth et al., 2023). The present contribution is narrower: it follows aligned weights and permutation-invariant representations through training, tests whether the grouping survives alternative measures and gauges, and separates a change in target rule from a change in input images.

The early trajectories also connect two bodies of work that make different predictions salient. Neural-tangent and lazy-training analyses show how learning can remain close to initialization under particular scaling and width conditions (Jacot et al., 2018; Chizat et al., 2019). The lottery-ticket hypothesis instead emphasizes that a random initialization may contain sparse subnetworks that can train especially effectively (Frankle and Carbin, 2019). Neither framework is tested directly here. In particular, our supplementary sign-stability analysis (Supplementary Figure S10) only summarizes the realized trajectories and is descriptive; it neither identifies a sparse winning ticket nor demonstrates causal selection of an initial subnetwork. What the early experiment does show is that group differences absent at initialization can emerge through small, coordinated updates. Establishing which theoretical regime best explains those updates will require targeted experiments rather than endpoint analogy.

### 4.9 Relation to biological convergence and the attractor hypothesis

Comparisons between biological and artificial systems provide a broader reason to ask whether common constraints produce common internal organization. Face representations in human cortex and deep networks can exhibit related geometries (Grossman et al., 2019); task-optimized artificial systems can recover motifs found in biological olfactory circuits (Wang et al., 2021); and recent work has framed such cross-system parallels as a form of brain–machine convergent evolution (Simony et al., 2024). The traffic can also run in the other direction: connectome structure from a living organism can guide the design of artificial networks (Bardozzo et al., 2024). Together, these studies make constraint-driven similarity a productive scientific question.

Our experiment operates at a different level. It compares independently trained artificial networks, not species, brains, or populations subject to inheritance and selection. Their trajectories were generated by gradient-based optimization under an explicit loss. Terms such as convergent development or canalization may therefore be closer analogies than biological evolution. We use the evolutionary perspective to motivate questions about repeated solutions under shared constraints, not to claim that the process studied here is evolutionary or that similarity between weight matrices establishes biological convergence.

The results nevertheless give the attractor idea a more concrete foundation as a hypothesis. Independent trajectories did not collapse onto one point, but they repeatedly entered task-conditioned regions after known symmetries were removed; changing the target rule redirected this organization, and the separation began early in training. These are the patterns one would want to explain with an algorithmic-attractor account. They are not sufficient to demonstrate one. In a dynamical sense, an attractor requires stability and a tendency to return after perturbation, together with evidence about its basin of attraction. None of those properties was tested here.

The target-rich landscape hypothesis concerns access to useful solution regions; the algorithmic-attractor hypothesis additionally concerns their dynamical stability. Accessible regions need not restore perturbed trajectories, and a large set of useful solutions need not form an attractor. The two hypotheses therefore require distinct tests even though both are motivated by the observed organization of training trajectories.

A precise future hypothesis is therefore that training defines task-conditioned stable sets in a quotient space of parameters, representations, or functions, rather than single preferred weight vectors. This formulation makes the speculation testable. If such a set acts as an algorithmic attractor, perturbing a trained network should lead trajectories back toward the same equivalence class; restarts from different nearby states should enter the same region; and changing the task should redirect trajectories toward a different region in a reproducible way. Failure of these restoring and basin-level predictions would favor the simpler explanation that we observed a structured distribution of optimization endpoints without attractor dynamics.

### 4.10 Limitations

Several limits follow directly from the scope of the evidence. All main networks were small multilayer perceptrons with one hidden ReLU layer, fixed zero biases, and the same width. The three main problems were related, low-resolution, grayscale classification datasets, and most experiments used one SGD configuration. These choices made exhaustive pairwise analyses and exact one-layer matching possible, but they do not establish that the same structure occurs in deep convolutional, recurrent, attention-based, or biologically constrained networks. They also leave open how optimizer, regularization, width, data augmentation, and training duration alter the effect.

The task control held images, input weights, and sample order fixed, but identity and parity required output layers of different sizes. It therefore isolates a target-rule contribution for *W*_0_ and hidden representations, not for the entire network. Alpha remains a sign-based, gauge-dependent dissimilarity outside the tested canonical convention, and pairwise matching need not yield a globally cycle-consistent labeling across many networks. CKA supplies an important representation-level check, but neither CKA nor activation correlation proves equality of computations.

Finally, we measured trajectories and endpoints, not basin geometry. We did not test loss barriers under interpolation, restoring responses to perturbations, repeated restarts from controlled neighborhoods, or switching between tasks during training. The supplementary sign-stability analysis is descriptive and does not supply these missing causal tests. Accordingly, the study does not demonstrate a dynamical attractor, a shared algorithm, deep-network generality, or an evolutionary process.

### 4.11 Outlook

The present limits suggest a direct sequence of experiments. The first step is to test whether the same dataset- and task-dependent grouping survives in deeper convolutional and recurrent networks, across optimizers and widths, and on tasks that differ in rule while sharing both inputs and output dimensionality. Strong permutation-aware baselines should be combined with cycle-consistent multi-network alignment so that a population of models can be placed in a common coordinate system rather than matched only pairwise. Functional interpolation and model-merging tests could then ask whether structural proximity corresponds to connected low-loss regions.

To test the target-rich landscape hypothesis, multiple initializations should be compared with independently sampled task-compatible solution sets using both raw and symmetry-adjusted distances under a specified normalization. Neighboring initializations and trajectories branched from common intermediate states can test whether nearby starts reach the same solution class and how the objective redirects them. The number or density of distinct task-compatible classes, defined at a stated resolution after removing symmetry copies, should then be related to early learning speed. Such measurements would test local accessibility directly rather than infer it from symmetry counts or small observed displacement alone.

The more demanding step is to test the attractor hypothesis itself. Networks can be perturbed at controlled distances in parameter, representation, and function space and then retrained under the same objective. Repeated restarts can map which initial states return to the same task-conditioned region and which cross a boundary. Branching trajectories from shared checkpoints while changing only the target rule would reveal whether the inferred basin follows the task rather than the initialization. These experiments should track trajectories in symmetry-adjusted spaces and define return before examining the data.

Such tests would turn the attractor perspective from an explanatory image into a falsifiable dynamical account. Evidence of robust return, basin boundaries, and task-directed switching would support the idea of algorithmic attractors as stable equivalence classes of learned organization. Their absence would be equally informative, indicating that reproducible clustering can arise without attractor dynamics. Either outcome would connect the careful comparison of artificial networks to the broader question that motivated this study: how strongly do shared computational constraints restrict the internal solutions available to a learning system?

## 5 Conclusion

Training did more than increase classification accuracy. Across independent initializations, it produced dataset- and task-dependent clusters in permutation-aligned weights and hidden representations. Dataset-dependent differentiation emerged through small early changes, persisted across several measures and controls, and remained visible when a stronger matching method was used; the separate fixed-input task control demonstrated a target-rule contribution. Our independently developed PIF-Hungarian procedure recovered functionally meaningful neuron correspondences, although Git Re-Basin provided the stronger alignment. Together, these results show that shared inputs and objectives restrict the internal solutions found by the networks, without forcing them to one weight configuration or establishing that they implement an identical algorithm.

The target-rich landscape hypothesis offers a possible account of early learning through small coordinated updates: task-compatible solution sets could be locally accessible after accounting for symmetry and redundancy. This accessibility has not been measured and should be distinguished from attraction. The repeated arrival of independent trajectories in task-conditioned regions makes an attractor perspective scientifically plausible, but the present evidence remains a starting point rather than a demonstration of attractor dynamics. The decisive next question is whether these regions behave as stable equivalence classes: whether perturbed or restarted networks return to them, and whether changing the task redirects that return. Framed in this testable way, the attractor hypothesis offers a route from the empirical clustering shown here toward a more general account of how learning constraints shape the internal organization of artificial neural networks.

## Data Availability

The raw data supporting the conclusions of this article will be made available by the authors, without undue reservation.

## Supplementary material

Supplementary Material Training diagnostics, complete layer-by-metric analyses, task-control results, representational similarity, ReLU-gauge checks, early-training trajectories, matching validation, descriptive sign stability, and a synthetic implementation sanity check.
