# Supplementary material for: Task-dependent clustering in permutation-aligned neural network weights
Source: Front Cognit. 2026 Oct 26;5:1960468. doi: 10.3389/fcogn.2026.1960468 (PMC13745664; doi:10.3389/fcogn.2026.1960468)
Supplement: Supplementary file 1 [file Data_Sheet_1.pdf]

Supplementary Material

Contents

Scope of the Supplementary Information 2
S1 Training cohort and diagnostics 2
S2 Full layer-by-metric trajectories 4
S3 Run-level distributions and robustness 6
S4 Task-control details 7
S5 Alpha–CKA validation details 9
S6 ReLU-rescaling stress test 11
S7 High-resolution SGD trajectories 13
S8 Matching trajectories and implementation checks 14
S9 Activation-based matching validation 16
S10 Descriptive sign-stability analysis 17
S11 Synthetic matching implementation sanity check 19

Scope of the Supplementary Information

This document reports the analyses that support, qualify, or extend the main results. Pair values are displayed to show the full structure of the data, but they are not treated as independent observations because each network occurs in multiple pairs. Unless stated otherwise, confidence intervals were therefore calculated by deleting complete training runs within dataset strata. The supplementary analyses describe dataset- and task-dependent organization of weights and representations; they do not establish an identical learned algorithm, a dynamical attractor, or an evolutionary process.

S1 Training cohort and diagnostics

Test accuracy and cross-entropy loss were read from the stored evaluation history of every network at every checkpoint. Thin trajectories represent individual training runs; thick trajectories are dataset means. Final-checkpoint distributions retain the complete run-level variation and are shown descriptively without treating checkpoints as independent observations.

All 30 networks learned their classification problem, and no run showed a failed trajectory. At the final checkpoint, mean test accuracy was 97.37% for MNIST, 86.37% for Fashion-MNIST, and 85.99% for KMNIST (Table S1). Variation among the ten runs in each group remained small. KMNIST provided the only notable qualification: its accuracy stabilized while cross-entropy loss increased late in training, consistent with increasing confidence on the remaining errors rather than declining classification accuracy.

Dataset	Runs	Accuracy, mean	Accuracy, s.d.	Loss, mean
MNIST	10	0.9737	0.0024	0.1127
Fashion-MNIST	10	0.8637	0.0047	0.4046
KMNIST	10	0.8599	0.0070	0.7992

Table S1: **Final training performance.** Values summarize independent runs at checkpoint E20; s.d., standard deviation across runs.

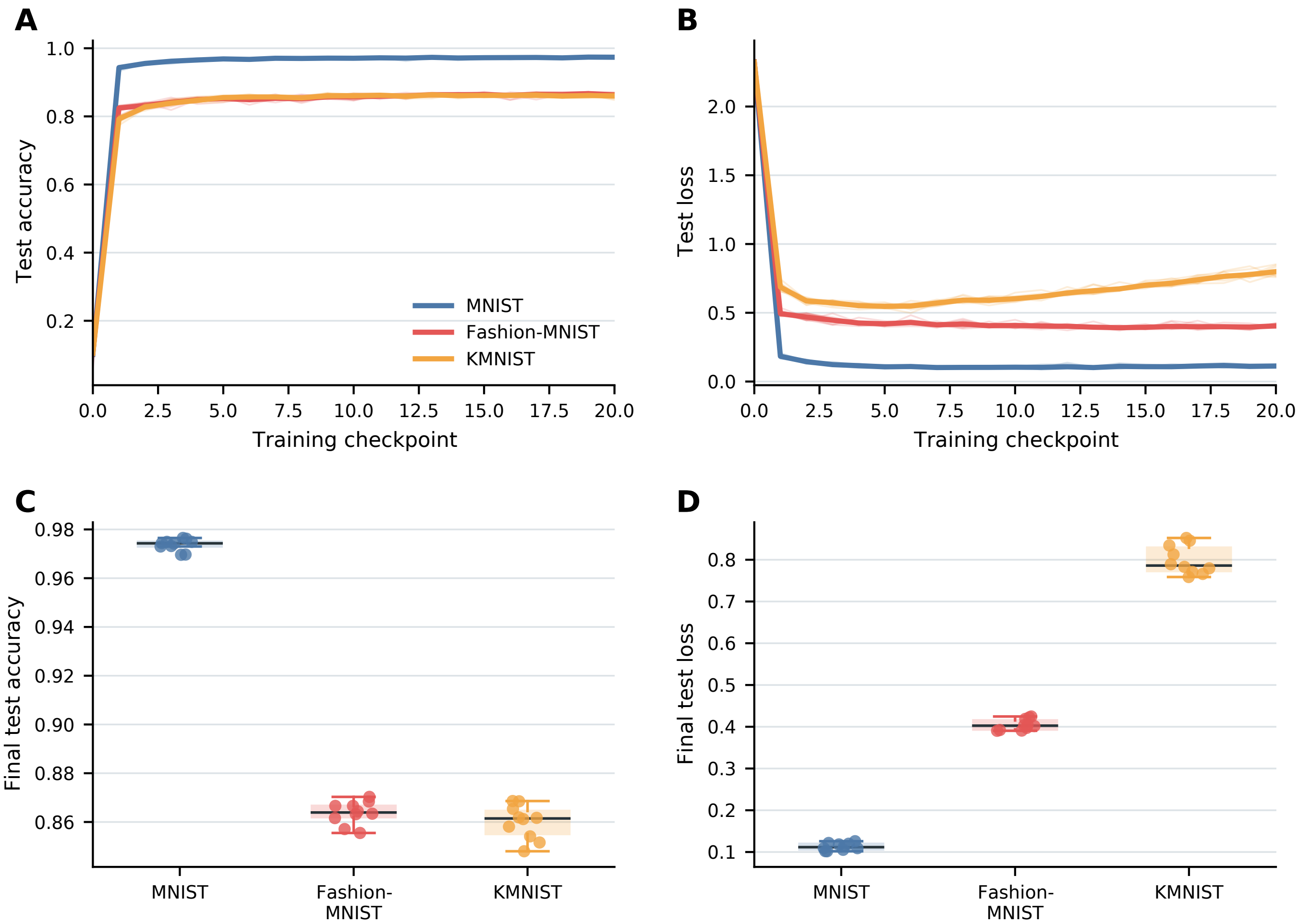

Figure S1: **Training converges reliably across datasets and independent runs. (A)**, Test-accuracy trajectories for 30 independent networks (ten per dataset) trained on MNIST, Fashion-MNIST, or KMNIST. Thin lines show individual runs and thick lines show dataset means. **(B)**, Corresponding test-loss trajectories. **(C)**, Run-level distributions of final test accuracy. **(D)**, Run-level distributions of final test loss. Each point represents one complete training run; no pairwise inferential statistics are used. The persistent rise of KMNIST loss despite stable accuracy reflects increasing confidence on the remaining errors and does not indicate a failure of accuracy convergence.

S2 Full layer-by-metric trajectories

For each checkpoint, Euclidean dissimilarity, SND, and raw Alpha were summarized separately for W_0, W_1, the equal-weighted mean of both layers, and the parameter-count-weighted mean. Within- and between-dataset estimates and their confidence intervals were obtained with the dataset-stratified delete-one-run jackknife defined in the main Methods.

Every layer-by-measure contrast was positive at E20 (Table S2). The relative size of the layer effects nevertheless depended strongly on the measure. Euclidean dissimilarity and SND gave larger absolute contrasts in W_1, whereas raw Alpha gave a much larger contrast in W_0. Parameter-count weighting assigned 98.74% of the aggregate to W_0 and therefore closely followed that layer. The robust conclusion is the direction of the within-/between-dataset separation; claims about the magnitude or geometry of the separation remain measure-dependent.

Measure	View	Between − within	95% confidence interval
Euclidean	W_0	0.02785	[0.02748, 0.02823]
Euclidean	W_1	0.09751	[0.09536, 0.09966]
Euclidean	Layer-balanced	0.06268	[0.06156, 0.06381]
Euclidean	Size-weighted	0.02873	[0.02836, 0.02911]
SND	W_0	0.08864	[0.08747, 0.08981]
SND	W_1	0.17724	[0.17428, 0.18021]
SND	Layer-balanced	0.13294	[0.13125, 0.13463]
SND	Size-weighted	0.08975	[0.08859, 0.09092]
Alpha	W_0	0.29380	[0.29010, 0.29749]
Alpha	W_1	0.01793	[0.01644, 0.01942]
Alpha	Layer-balanced	0.15586	[0.15369, 0.15803]
Alpha	Size-weighted	0.29032	[0.28667, 0.29397]

Table S2: **Final raw weight-dissimilarity contrasts.** Positive values indicate greater dissimilarity between than within datasets. Matching was performed separately for each measure. Intervals use the dataset-stratified delete-one-run jackknife.

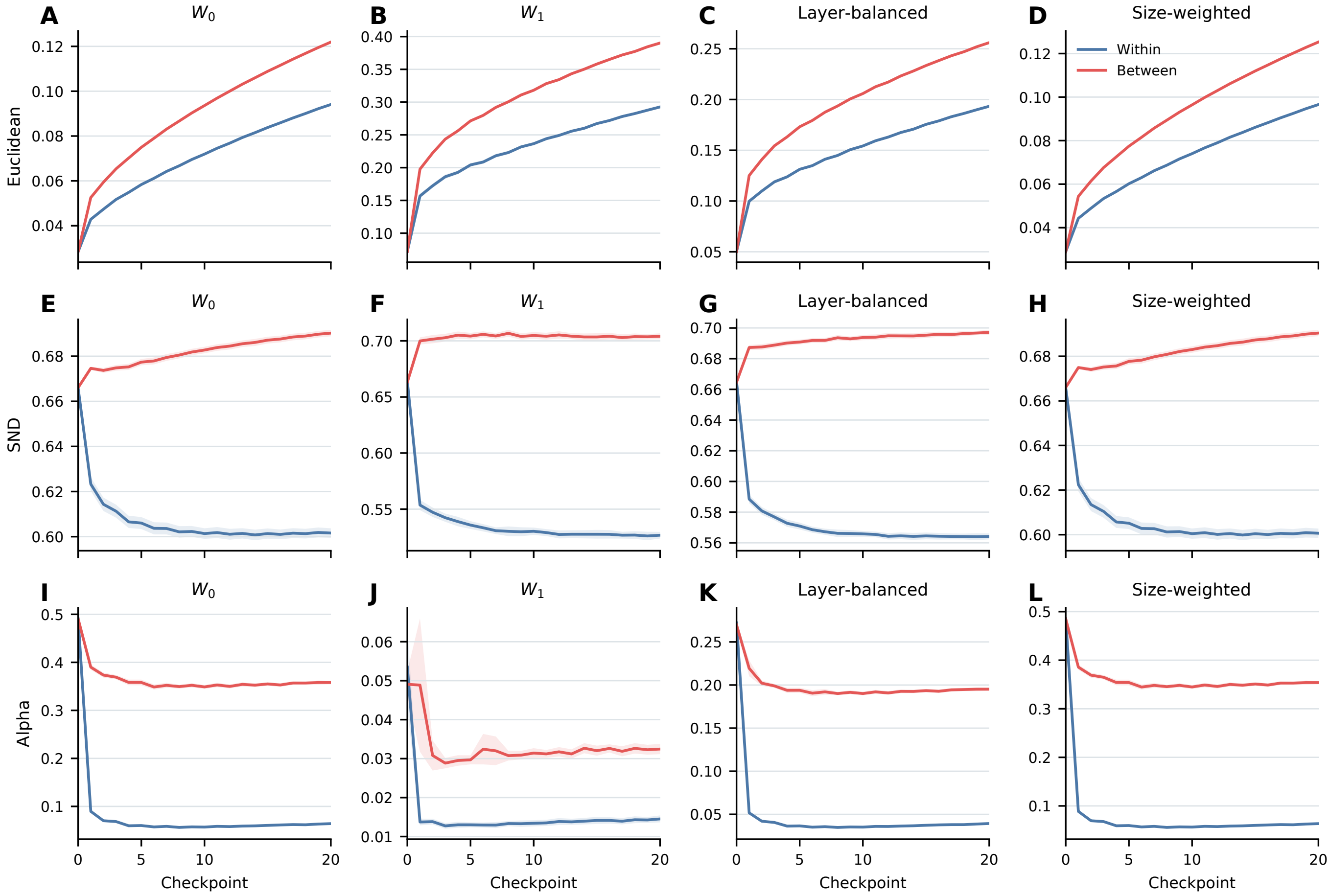

Figure S2: **Weight-space separation is consistent across metrics, layers and training checkpoints.** Assignments use the corresponding main-cohort profile cost, with PIF initialization; Alpha here is raw rather than canonically gauged. Rows show Euclidean dissimilarity (**A**–**D**), SND (**E**–**H**), and Alpha with $\alpha = 2$ (**I**–**L**). Columns show W_0, W_1, the layer-balanced mean, and the parameter-count-weighted mean. Blue curves represent 135 network pairs trained on the same dataset and red curves represent 300 pairs trained on different datasets. Lines show group estimates and shaded regions show 95% confidence intervals obtained by a dataset-stratified delete-one-run jackknife across 30 complete training runs; pair counts are descriptive. The scale differs among panels so that the temporal development of each measure remains visible.

S3 Run-level distributions and robustness

For each network at the final checkpoint, we calculated its mean dissimilarity to the other runs from the same dataset and to all runs from the other datasets. Their difference is the run-level contribution to the global between-minus-within contrast. These contributions expose whether an aggregate result is driven by a small subset of networks.

All 30 run contributions were positive for Alpha in both layers, and the same direction held for every plotted measure and aggregation. The contributions varied in size, particularly across datasets for W_0, but no isolated run determined the pooled result. The plotted points are useful for showing replication across complete runs; inferential uncertainty still comes from the stratified jackknife rather than from treating the 435 shared-network pairs as independent samples.

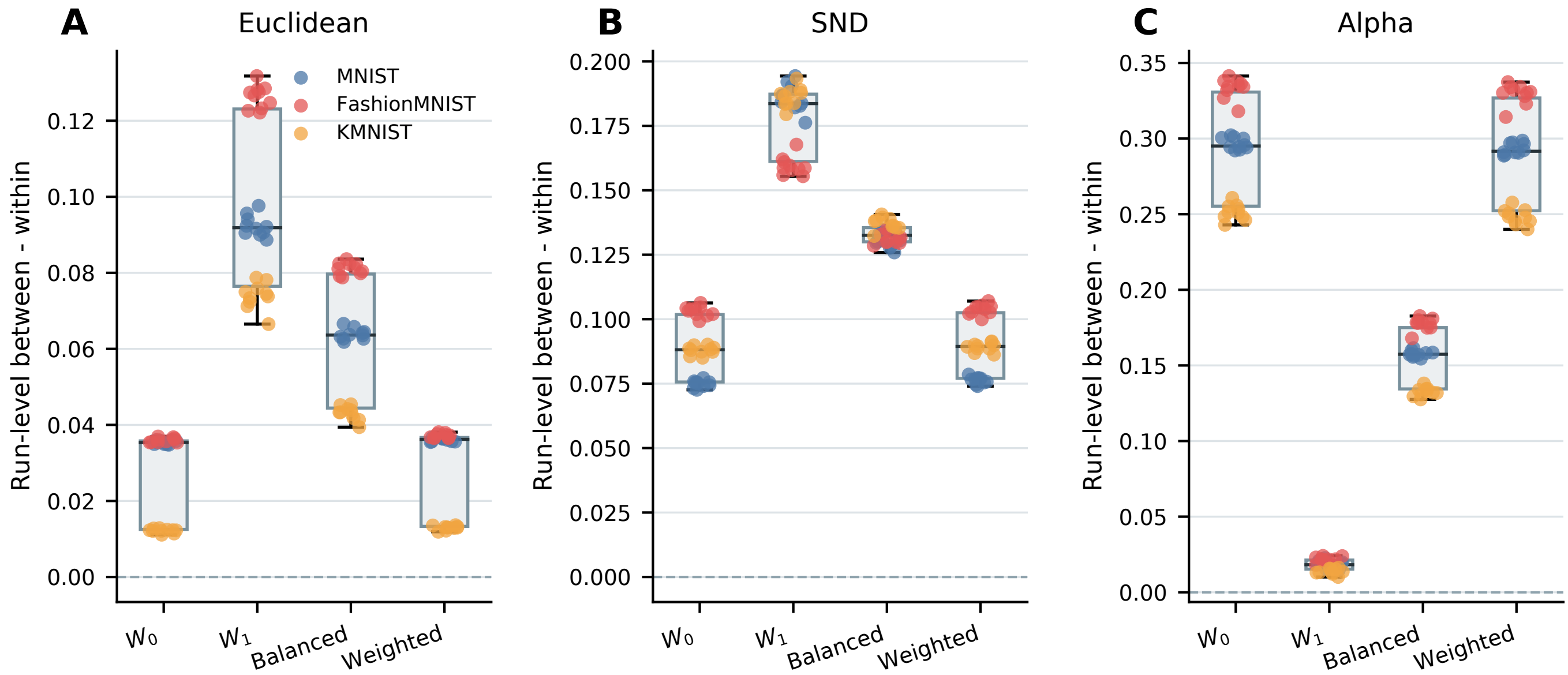

Figure S3: **Dataset-dependent contrasts replicate across individual training runs.** Run-level between-minus-within contributions at the final checkpoint for Euclidean dissimilarity (**A**), SND (**B**), and Alpha (**C**). Within each panel, results are shown for W_0, W_1, the layer-balanced mean, and the parameter-count-weighted mean. Each point is one of 30 complete training runs; colors identify the training dataset. Boxes summarize the pooled distribution across runs. Positive contributions across datasets and analysis views show that the group separation is not attributable to a small number of influential networks.

S4 Task-control details

The paired task control used the same MNIST images, identical input-layer initialization within each seed block, and the same image order, while changing only the prediction rule between digit identity and parity. Uncertainty was estimated by deleting an entire paired seed block. Group-specific endpoint means distinguish within-identity, within-parity, same-seed cross-task, and different-seed cross-task comparisons.

Both tasks were learned reliably, reaching mean final accuracies of 97.25% for identity and 98.60% for parity. Although the paired networks began with bitwise identical W_0 matrices, training produced positive between-task contrasts under all three weight measures and CKA (Table S3). The same-seed cross-task Alpha mean was 0.07857, close to 0.08153 for cross-task comparisons with different seeds. The task effect therefore cannot be explained by different random initializations. Alpha uses raw W_0 and incoming-weight-only Hungarian assignment; the implemented whole-network Git Re-Basin objective is not directly applicable across the different output dimensions. Because the output dimensions differed, this control supports a target-rule effect for W_0 and the hidden representation, not a whole-network comparison.

Measure	Between-task − within-task	95% confidence interval
Euclidean W_0	0.01152	[0.01114, 0.01191]
SND W_0	0.02602	[0.02503, 0.02702]
Alpha W_0	0.04452	[0.04110, 0.04793]
Hidden 1 − CKA	0.13658	[0.12445, 0.14872]

Table S3: **Final task-control contrasts.** Intervals were obtained by deleting complete paired seed blocks.

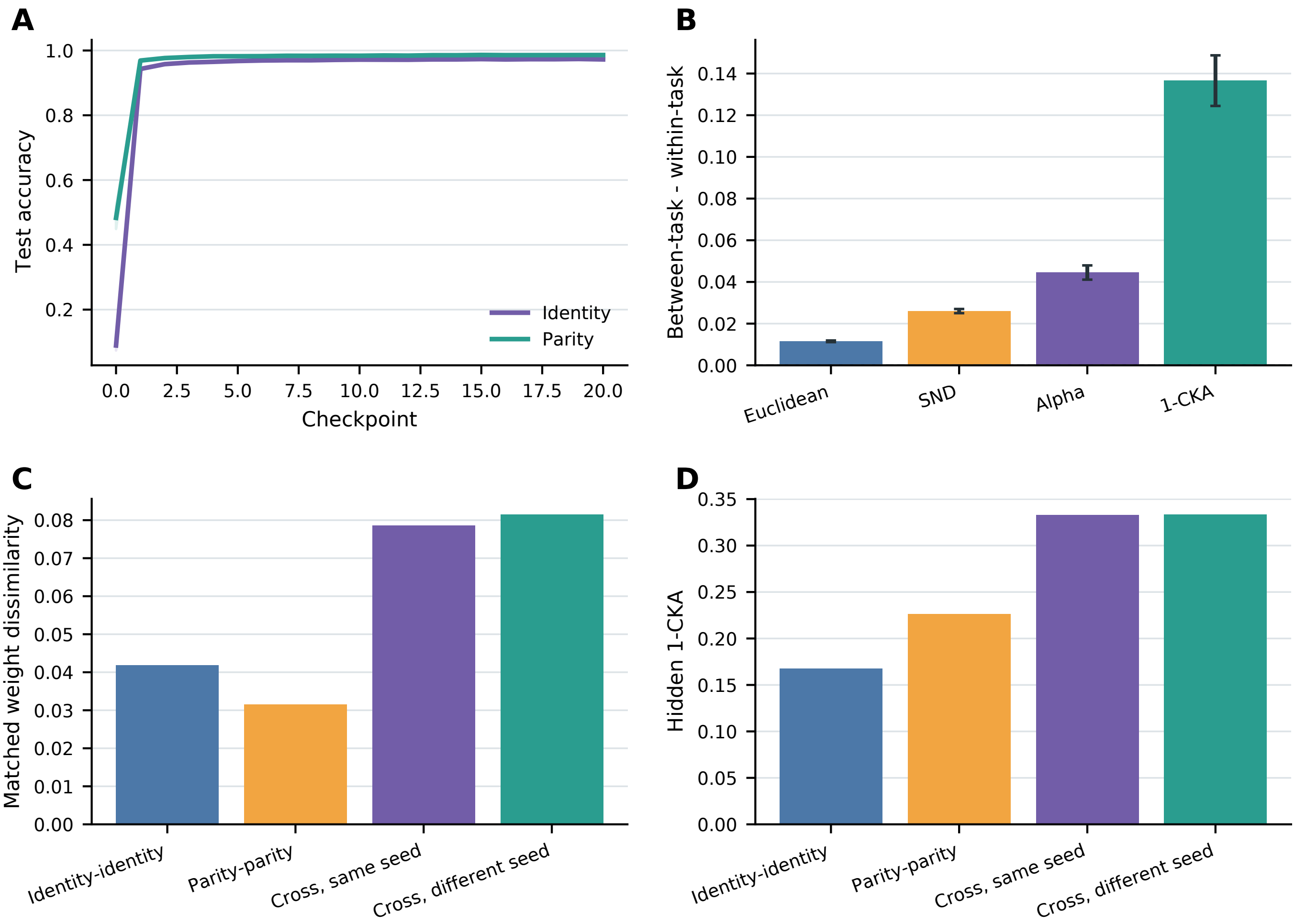

Figure S4: **Task-dependent separation is robust across weight measures and seed comparisons. (A)**, Mean test-accuracy trajectories for ten paired seed blocks under the identity and parity objectives; shaded regions show 95% Student-t intervals across paired runs. **(B)**, Final between-task-minus-within-task contrasts for Euclidean dissimilarity, SND, matched W_0 Alpha, and hidden $1 - \text{CKA}$; whiskers show 95% paired-block jackknife confidence intervals. **(C,D)**, Final subgroup means for matched weight dissimilarity and hidden-representation dissimilarity. The independent unit for inference is one complete paired seed block. Cross-task networks separate even when they share the same input images, initialization seed, and training order.

S5 Alpha–CKA validation details

Pearson and Spearman correlations were calculated between pairwise raw Alpha dissimilarity and hidden $1 - \text{CKA}$ at each sampled checkpoint. Confidence intervals were estimated by deleting complete runs, rather than treating the many network pairs as independent. Correlations were also calculated separately within and between dataset groups to reveal correlations induced by the group separation itself.

At E20, Alpha and CKA produced similarly strong dataset contrasts, but their pairwise association depended on which pairs were included. The pooled Pearson correlation was 0.9269, fell to 0.6524 within datasets, and was only 0.1071 between datasets (Table S4). Thus, the high pooled value mainly reflects agreement on the broad dataset grouping. Alpha and CKA are complementary measurements and should not be treated as interchangeable rankings of individual network pairs.

Pairs	Pearson r	95% confidence interval	Spearman ρ	95% confidence interval
All	0.9269	[0.9162, 0.9377]	0.6916	[0.6384, 0.7448]
Within dataset	0.6524	[0.5268, 0.7780]	0.4587	[0.4150, 0.5024]
Between datasets	0.1071	[−0.0823, 0.2965]	0.1091	[−0.0517, 0.2699]

Table S4: **Final association between matched W_0 Alpha and hidden $1 - \text{CKA}$.** Confidence intervals delete complete runs.

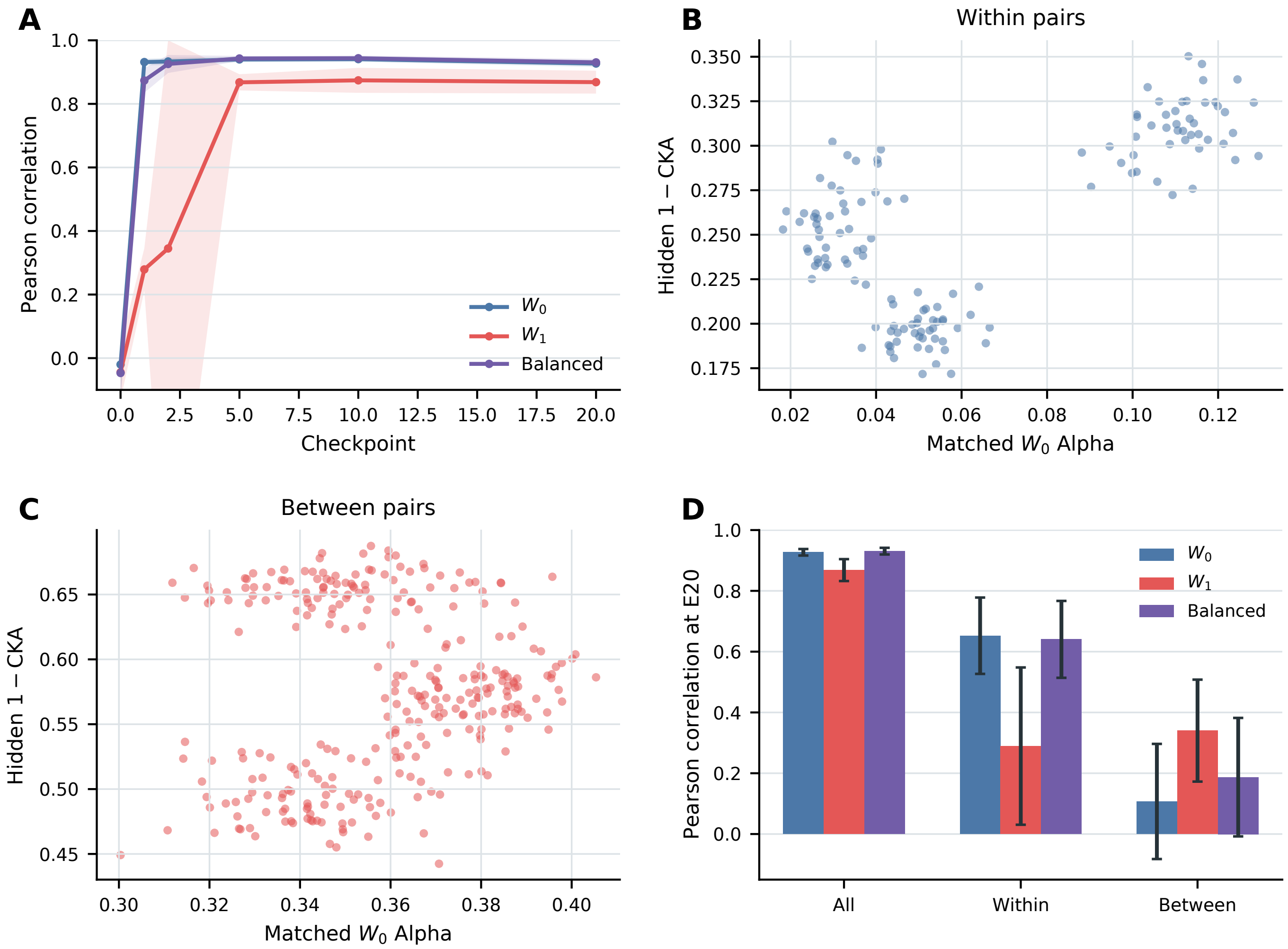

Figure S5: **Alpha and CKA agree on group structure but differ in pairwise detail. (A)**, Pearson correlations over training between hidden $1 - \text{CKA}$ and matched Alpha for W_0, W_1, and the layer-balanced mean. Lines show estimates and shaded regions show 95% run-level jackknife confidence intervals across 30 independent networks. **(B,C)**, Final-checkpoint relationship between matched W_0 Alpha and hidden $1 - \text{CKA}$ for 135 within-dataset and 300 between-dataset pairs; pair values are descriptive. **(D)**, Final Pearson correlations for all pairs and for the two pair groups separately. The strong pooled association is largely produced by their common dataset separation; conditional pairwise correlations are weaker and less stable.

S6 ReLU-rescaling stress test

Positive rescaling of a hidden neuron can multiply its incoming weights and divide its outgoing weights without changing the represented function. We applied deterministic neuronwise rescalings spanning progressively wider multiplicative envelopes. Raw Alpha was recalculated after rescaling, while the canonical gauge was checked by comparing recovered weights elementwise. Logits and predicted classes on the fixed probe set verified functional invariance.

The transformations preserved every predicted class and changed logits by at most 4.26×10^{-14}, yet raw Alpha changed materially (Table S5). At the strongest envelope, the layer-balanced contrast retained 64.7% of its original value but remained positive. Canonicalization reduced the maximum weight difference between transformed and untransformed networks to 1.55×10^{-15} and yielded a final layer-balanced contrast of 0.15901 [0.15535, 0.16266]. These checks preserve the qualitative dataset result while showing that raw Alpha values are not intrinsic properties of the represented function.

C	Mean absolute change	Correlation with original	Dataset contrast	Fraction retained
1.00	0.00000	1.000	0.15586	100.0%
1.25	0.00797	0.994	0.14784	94.9%
1.50	0.01940	0.986	0.13314	85.4%
2.00	0.03507	0.968	0.11379	73.0%
4.00	0.04483	0.935	0.10145	65.1%
8.00	0.04462	0.911	0.10084	64.7%

Table S5: **Sensitivity of raw layer-balanced Alpha at E20.** The envelope C bounds the neuronwise positive rescaling factors. All transformations preserve the network function.

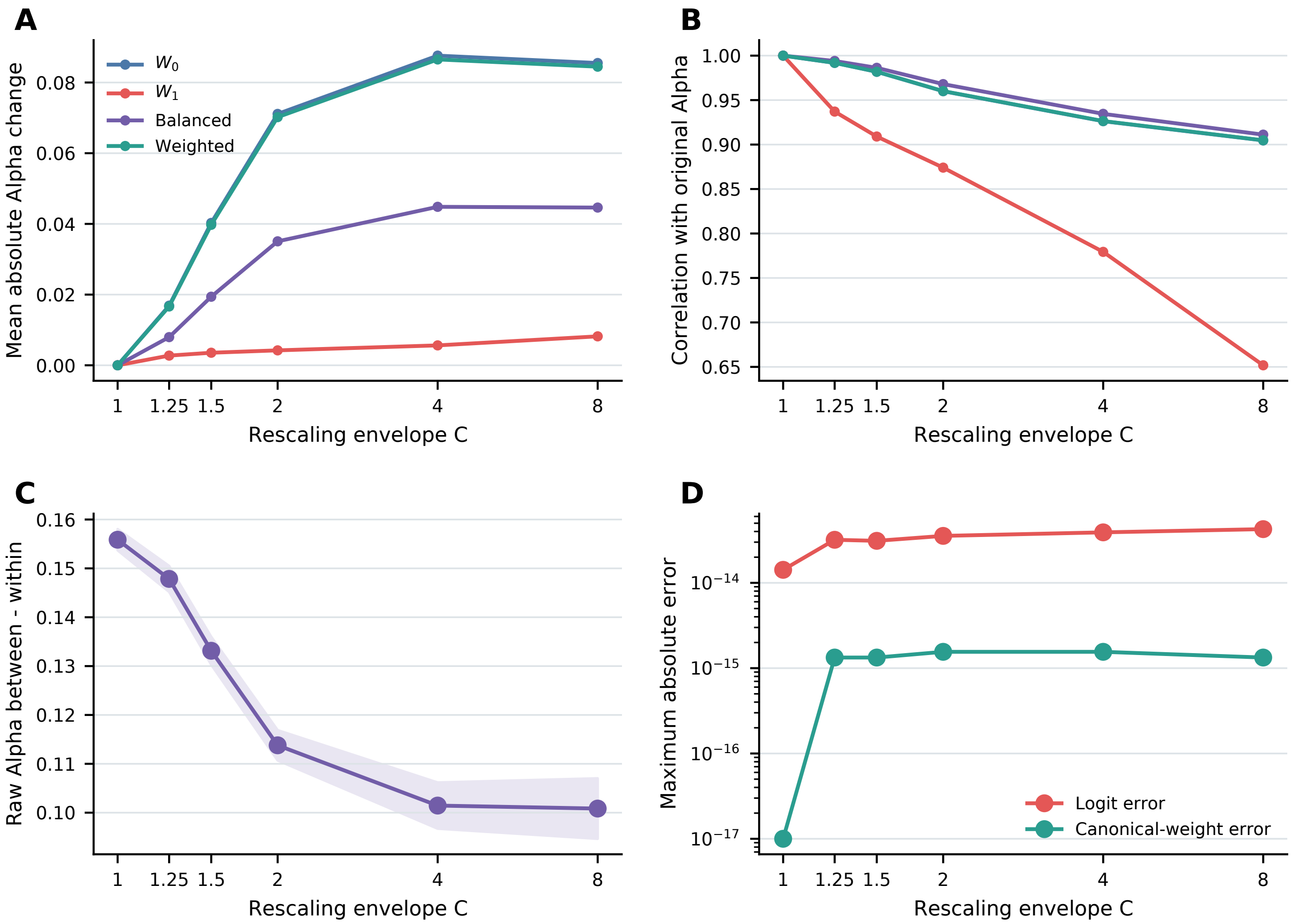

Figure S6: **Canonicalization removes ReLU-rescaling freedom from Alpha comparisons.** **(A)**, Mean absolute change in raw pairwise Alpha as the rescaling envelope widens, shown by layer and aggregation rule for all 435 pairs among 30 networks. **(B)**, Correlation of rescaled raw Alpha with its untransformed value. **(C)**, Dataset contrast of raw layer-balanced Alpha with 95% confidence intervals from a dataset-stratified delete-one-run jackknife across complete runs. **(D)**, Maximum absolute logit error and maximum elementwise canonical-weight error over 180 network-by-envelope transformations. Network predictions agreed exactly in all checks, and canonicalization recovered the same weights to numerical precision even though raw Alpha values changed materially.

S7 High-resolution SGD trajectories

The early-SGD experiment stored checkpoints densely from initialization through the first two epochs. For every measure, onset was defined before inspection of the results as the first post-initialization checkpoint whose complete run-level confidence interval for the between-minus-within contrast lay above zero. The nonuniform step axis is displayed as $\log_2(s+1)$ so that the earliest updates remain visible.

Dataset-dependent representation structure was detected after two updates, and every weight measure crossed the same prespecified criterion by update four (Table S6). Canonical Alpha had an onset contrast of 0.1322 [0.1072, 0.1571], while the CKA contrast at update two was 0.0860 [0.0655, 0.1066]. In contrast, the mean functional 90% point occurred at update 64. The result therefore rejects the tested calibration-then-consolidation ordering. It does not establish a lazy-training or neural-tangent-kernel regime, which would require direct linearization and kernel-stability tests.

Prespecified criterion	First qualifying update
Hidden linear CKA contrast	2
Raw Alpha contrast	2
Euclidean contrast	4
SND contrast	4
Canonical Alpha contrast	4
Mean functional progress $\geq$ 90%	64

Table S6: **Onset of early functional and structural changes.** Structural onsets require the complete run-level 95% confidence interval to lie above zero.

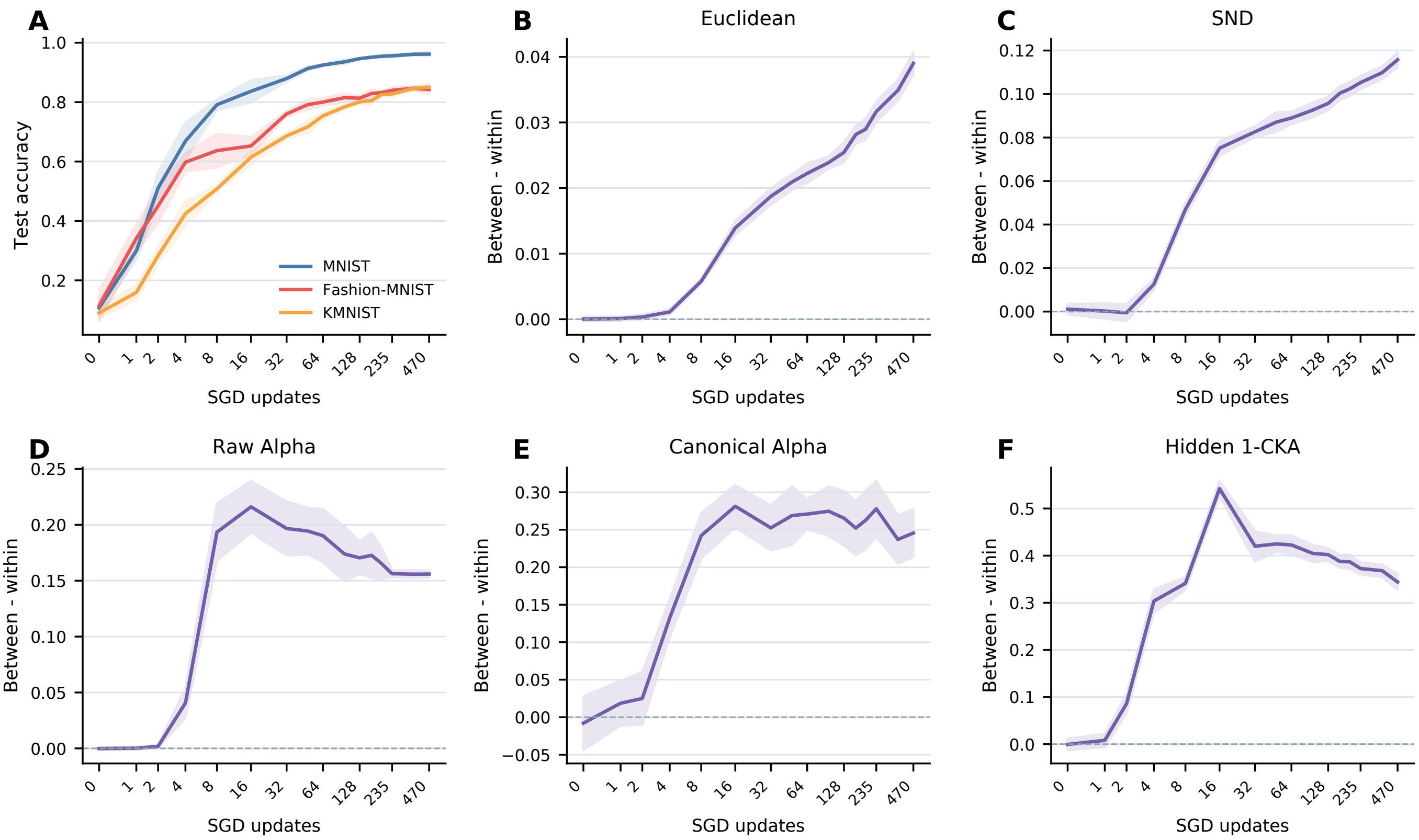

Figure S7: **Multiple measures resolve the onset of dataset-specific structure during early SGD. (A)**, Test-accuracy trajectories for five independent runs per dataset (15 total). **(B–F)**, Between-minus-within dataset contrasts for Euclidean dissimilarity, SND, raw Alpha, canonical Alpha, and hidden $1-$CKA across all 105 network pairs. Lines show estimates and shaded regions show 95% confidence intervals from a dataset-stratified delete-one-run jackknife; horizontal dashed lines mark zero contrast. Pair counts are descriptive and complete runs are the independent units. Representation-space separation was detectable first, followed closely by canonical weight-space separation, well before most of the early accuracy progress had accumulated.

S8 Matching trajectories and implementation checks

The unmatched order, PIF-Hungarian assignment, and exact one-hidden-layer Git Re-Basin assignment were evaluated at every checkpoint. Here the PIF-Hungarian label refers to the candidate-selected benchmark procedure documented in the main Methods: candidate selection uses parameter-count-weighted Alpha, whereas the displayed canonical Alpha endpoint is layer-balanced. It is distinct from the direct canonical-Alpha profile assignment in the principal analysis. Random-renumbering tests permuted the neuron indices of the second network, repeated each matching, and composed the inferred assignment back into the original indexing. Assignment overlap is the fraction of hidden neurons assigned identically by PIF-Hungarian and Git Re-Basin.

Git Re-Basin was strongest for every pooled E20 endpoint (Table S7). Its advantage was expected for weight cosine, which is closely related to its objective, but it also extended to canonical Alpha and activation correspondence. The two methods agreed on only 16.5% of assignments [15.7%, 17.3%], showing that their objectives selected different correspondences. Both methods returned exactly the same assignment after random neuron renumbering, and the resulting permutations preserved the network function to numerical precision. These are implementation and equivariance checks, not evidence that either assignment is a unique neuronal correspondence.

Endpoint	Unmatched	PIF-Hungarian	Git Re-Basin
Weight cosine, higher is better	0.0288	0.1774	0.2885
Weight RMSE, lower is better	0.1402	0.1288	0.1195
Canonical Alpha, lower is better	0.4780	0.2324	0.1496
Activation correlation, higher is better	0.0502	0.1314	0.2734

Table S7: **Pooled matching endpoints at E20.** The Alpha values are canonical and layer-balanced.

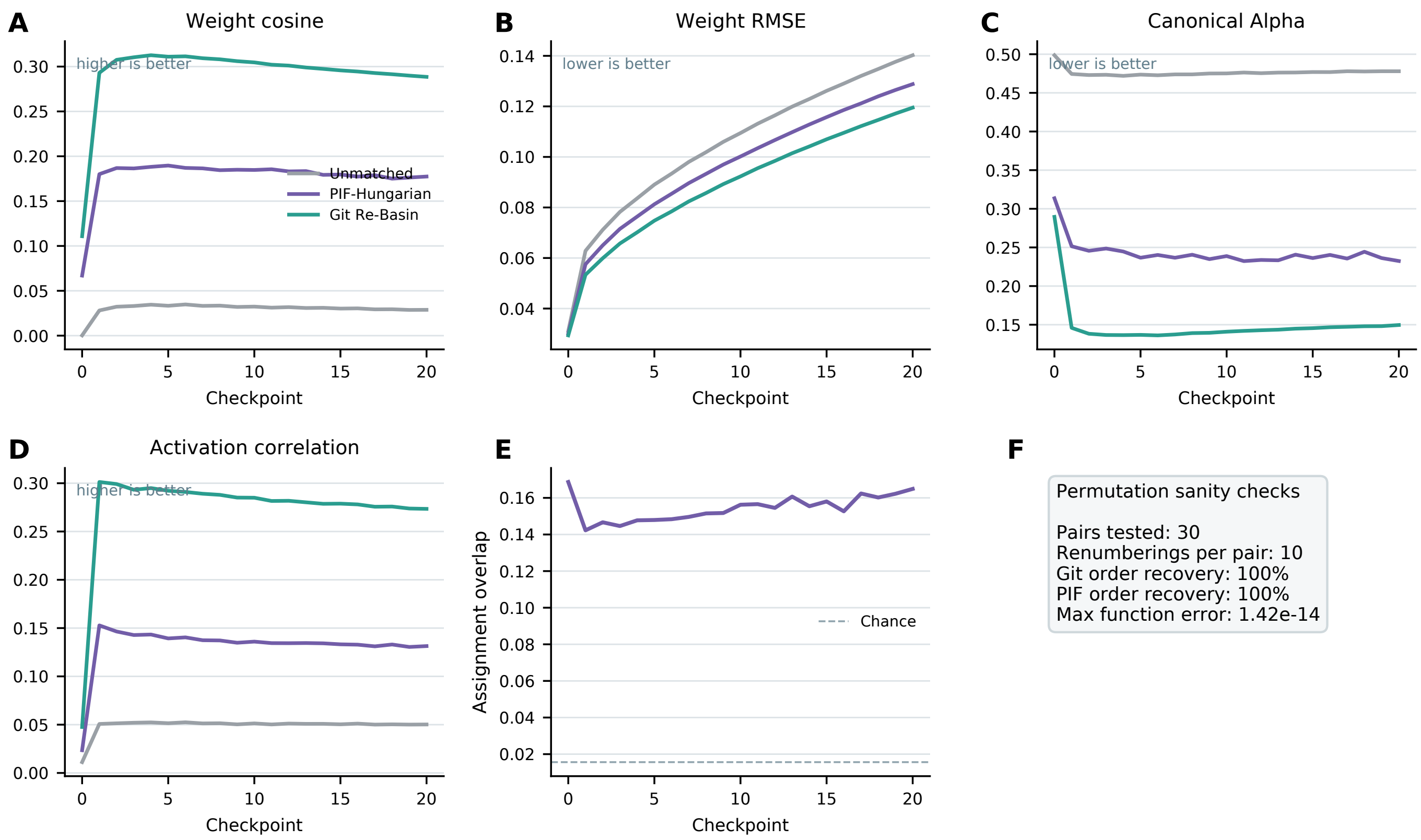

Figure S8: **Matching methods differ throughout training and remain invariant to neuron renumbering.** Pooled trajectories over all 435 pairs among 30 networks for raw weight-cosine similarity (**A**), raw weight RMSE (**B**), canonical Alpha dissimilarity (**C**), and independent activation correspondence (**D**). Lines show estimates and shaded regions show 95% confidence intervals from a dataset-stratified delete-one-run jackknife. **(E)**, Mean fraction of identical assignments produced by PIF-Hungarian and Git Re-Basin; the dashed line marks chance agreement. **(F)**, Prespecified sanity checks on 30 final-checkpoint pairs with ten random renumberings each. Both procedures recovered exactly the same assignments after random neuron renumbering, and applying either permutation preserved network function to numerical precision. Git Re-Basin was stronger throughout training, while the modest assignment overlap shows that the objectives identify substantially different correspondences.

S9 Activation-based matching validation

Activation correspondence was measured on the common probe set after centring each hidden-neuron activation vector. Because activations were not used in either weight-based assignment, the measure provides an independent assessment of functional correspondence. Endpoint distributions use individual pairs descriptively; inferential intervals reported in the main text remain based on complete-run deletion.

PIF-Hungarian matching increased pooled activation correlation by 0.0812 [0.0780, 0.0844] relative to the unmatched order. The improvement was strongest within datasets, where all 135 pairs improved and the mean correlation rose from 0.0655 to 0.3078. Among between-dataset pairs, the mean increase was smaller and 69.7% of pairs improved. Git Re-Basin improved every pair, exceeded PIF-Hungarian for every pair, and reached mean correlations of 0.4928 within and 0.1747 between datasets. The authors' procedure therefore has independently measurable functional value, but the established baseline is clearly stronger.

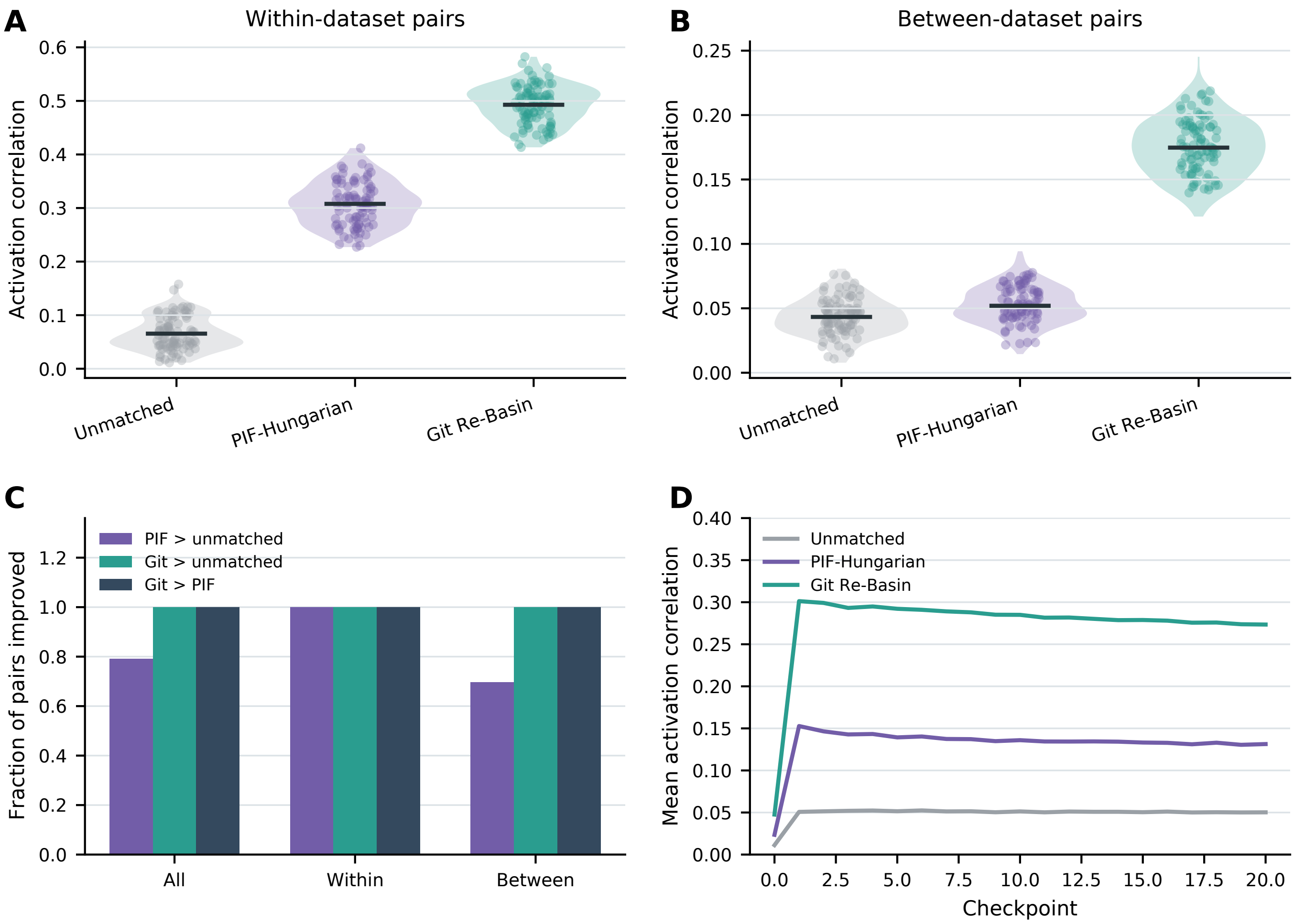

Figure S9: **Activation correspondence validates permutation-based neuron matching.** Distributions of final matched-neuron activation correlations for 135 within-dataset pairs (**A**) and 300 between-dataset pairs (**B**) in the unmatched, PIF-Hungarian, and Git-Re-Basin conditions. Horizontal segments mark distribution means; displayed pair values are descriptive because pairs share 30 networks. **(C)**, Fraction of pairs for which PIF-Hungarian exceeded the unmatched order, Git Re-Basin exceeded the unmatched order, or Git Re-Basin exceeded PIF-Hungarian. **(D)**, Pooled mean activation correspondence throughout training; uncertainty is estimated by a dataset-stratified delete-one-run jackknife. PIF-Hungarian consistently recovered functional correspondence within datasets, whereas Git Re-Basin produced the larger and more general improvement.

S10 Descriptive sign-stability analysis

For each run, layer, and checkpoint, sign retention relative to initialization was calculated as

$$S_\ell(e) = \frac{1}{|W_\ell|} \sum_{i,j} \mathbf{1}[\operatorname{sign} W_{\ell,ij}(e) = \operatorname{sign} W_{\ell,ij}(0)]\,. \quad (1)$$

The complementary sign-change fraction is $1 - S_\ell(e)$. This newly tabulated analysis is descriptive: it characterizes how much of the initialized sign pattern remains, but does not identify a causal mechanism or a privileged structural core.

Most sign changes occurred during the first recorded training interval and accumulated more gradually thereafter. At E20, mean retention in W_0 ranged from 0.5627 for KMNIST to 0.6719 for MNIST; the corresponding W_1 values ranged from 0.6900 to 0.7234 (Table S8). These layer- and dataset-dependent values describe the realized trajectories only. Because the analysis conditions on the observed trained weights and uses no displacement-matched or shuffled-label null model, it cannot identify a lottery ticket, show that useful structure was already present at initialization, or establish that sign retention caused the dataset clustering.

Dataset	W_0, E01	W_0, E20	W_1, E01	W_1, E20
MNIST	0.7925	0.6719	0.7986	0.7205
Fashion-MNIST	0.7227	0.6075	0.7631	0.6900
KMNIST	0.6996	0.5627	0.7913	0.7234

Table S8: **Mean sign retention relative to initialization.** Values are descriptive means over ten independent runs per dataset.

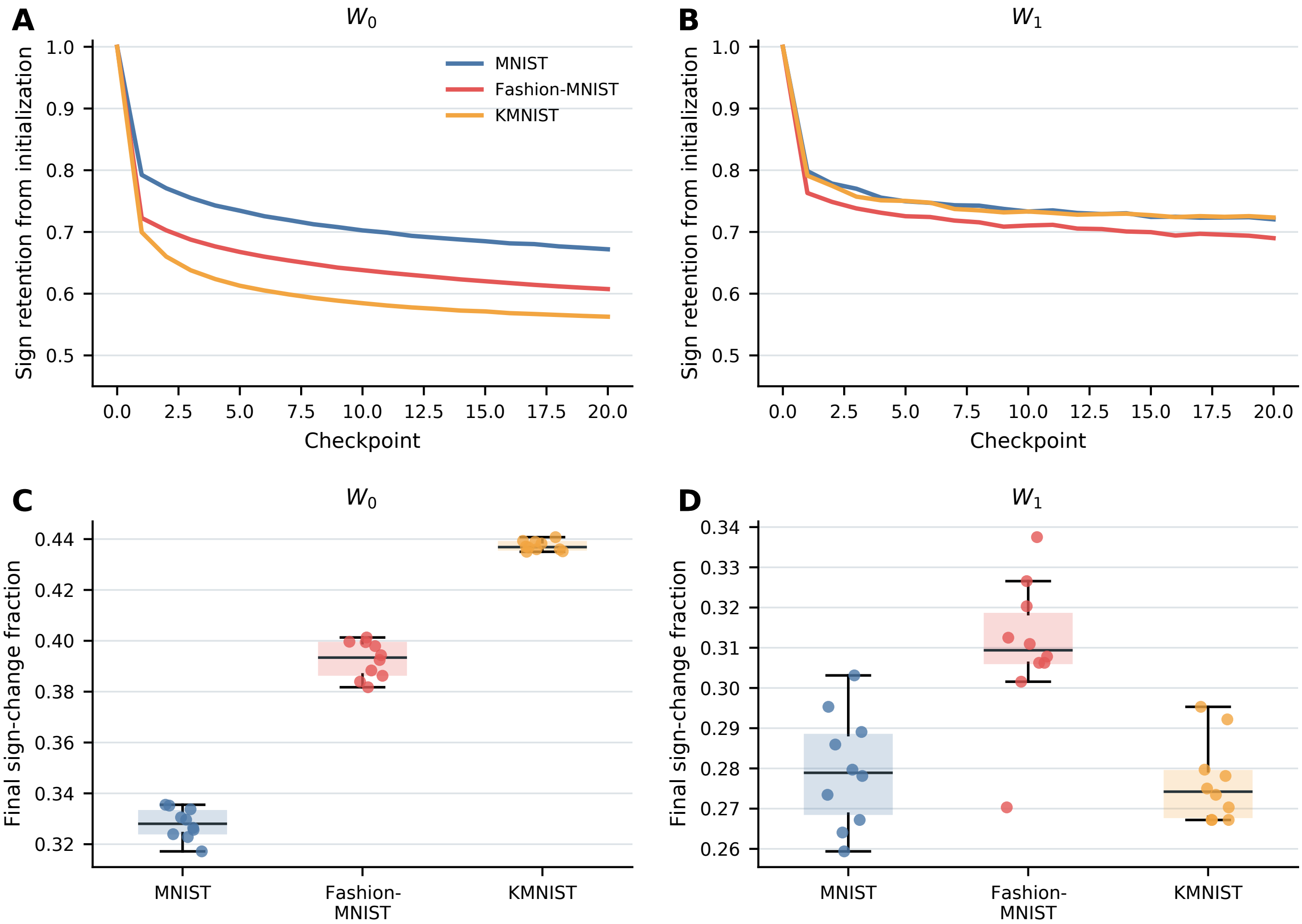

Figure S10: **Weight-sign retention is a descriptive feature of training trajectories.** Mean fraction of weights retaining their initial sign across training for W_0 (**A**) and W_1 (**B**), separated by dataset and averaged over ten independent runs per dataset. **(C,D)**, Run-level distributions of the complementary final sign-change fraction; each point represents one complete run and no inferential pairwise analysis is used. Sign patterns changed most rapidly during the earliest training interval and then evolved more gradually. Retention differed across datasets and layers, but these descriptive differences are not interpreted as evidence that sign preservation causes the observed dataset-dependent clustering.

S11 Synthetic matching implementation sanity check

The iterative multilayer implementation was also checked in a deliberately favorable synthetic setting. A random network with architecture $20 \to 12 \to 10 \to 8 \to 6$ was copied, its three hidden layers were permuted, and independent Gaussian noise with a standard deviation equal to 30% of that of each weight matrix was added. The noisy copy was then aligned to the original with Euclidean, SND, or Alpha profile costs. Matching reduced the corresponding full-network dissimilarities from 0.40174 to 0.08844, from 0.70260 to 0.26841, and from 0.44991 to 0.00008, respectively.

This construction has a known ground-truth correspondence and therefore tests whether the code can recover an imposed multilayer permutation in the presence of noise. It is not an independent training experiment, does not validate the zig-zag procedure on separately optimized deep networks, and does not support a claim of state-of-the-art matching. The activation-based comparisons of independently trained networks in Fig. S9 provide the more relevant functional validation for the empirical study.

Matching cost	Before matching	After matching
Euclidean	0.40174	0.08844
SND	0.70260	0.26841
Alpha, $\alpha = 2$	0.44991	0.00008

Table S9: **Synthetic multilayer implementation check.** The test evaluates recovery of a known imposed correspondence and should not be interpreted as evidence from independently trained deep networks.
